# Class I HDAC inhibition is a novel pathway for regulating astrocytic apoE secretion

**DOI:** 10.1371/journal.pone.0194661

**Published:** 2018-03-26

**Authors:** Erica Dresselhaus, James M. Duerr, Fabien Vincent, Emily K. Sylvain, Mercedes Beyna, Lorraine F. Lanyon, Erik LaChapelle, Martin Pettersson, Kelly R. Bales, Gayathri Ramaswamy

**Affiliations:** 1 Internal Medicine Research Unit, Pfizer, Cambridge, Massachusetts, United States of America; 2 Hit Discovery and Lead Profiling, Pfizer, Groton, Connecticut, United States of America; 3 Medicinal Chemistry, Pfizer, Groton, Connecticut, United States of America; 4 Medicinal Chemistry, Pfizer, Cambridge, Massachusetts, United States of America; Sungkyunkwan University, REPUBLIC OF KOREA

## Abstract

Despite the important role of apolipoprotein E (apoE) secretion from astrocytes in brain lipid metabolism and the strong association of apoE4, one of the human apoE isoforms, with sporadic and late onset forms of Alzheimer’s disease (AD) little is known about the regulation of astrocytic apoE. Utilizing annotated chemical libraries and a phenotypic screening strategy that measured apoE secretion from a human astrocytoma cell line, inhibition of pan class I histone deacetylases (HDACs) was identified as a mechanism to increase apoE secretion. Knocking down select HDAC family members alone or in combination revealed that inhibition of the class I HDAC family was responsible for enhancing apoE secretion. Knocking down LXRα and LXRβ genes revealed that the increase in astrocytic apoE in response to HDAC inhibition occurred via an LXR-independent pathway. Collectively, these data suggest that pan class I HDAC inhibition is a novel pathway for regulating astrocytic apoE secretion.

## Introduction

Apolipoprotein E (apoE), a 34 kDa lipid transport protein, plays an important role in lipid metabolism in both the periphery and the central nervous system (CNS). Specifically, in plasma, apoE is involved in clearance of triglyceride-rich and cholesterol-rich lipoprotein particles and reverse-cholesterol transport [1, 2]. Hepatocytes are the predominant cell type responsible for apoE in the plasma, though other peripheral cell types, such as macrophages and adipocytes, contribute. Astrocytes are the main apoE producing cells in the brain, where apoE has been suggested to play a role in homeostasis, neuroinflammation, neuronal repair and integrity, synaptogenesis, and clearance of amyloid-β (Aβ) [2–4]. Humans have three common apoE isoforms, namely, apoE2, apoE3 and apoE4, which differ only at amino acid positions 112 and 158. These amino acid differences have profound effects on the physical properties of apoE, including lipidation status and the ability to bind to receptors [1, 3, 5]. Additionally, apoE has been shown to have a strong genotype-dependent association with risk and age of onset for the development of sporadic and late-onset forms of Alzheimer’s disease (AD); apoE4 is associated with the highest risk whereas apoE2 has been suggested to be protective [3, 6, 7]. Interestingly, a genotype dependent effect on apoE level (apoE2 > apoE3 > apoE4) has been observed in human CSF and in mouse models expressing one of the three human apoE isoforms [8–10].

ApoE is located on chromosome 19 within a 44 kb gene cluster formed by apoE, apoC-I, apoC-II and apoC-IV genes [11]. Studies expressing human apoE gene fragments in mice showed that the proximal apoE gene promoter cannot direct gene expression by itself in the brain *in vivo* [12]. In fact, two cis-acting enhancer elements present downstream of the gene are required for astrocytic apoE expression [12]. The enhancers ME1 and ME2 are located 2.2 kb and 15 kb downstream of the apoE gene, respectively. ME2 shares 95% nucleotide sequence identity with ME1 and is thought to be a result of duplication of the ME1 sequence [13]. Sequence analysis suggests several transcription factor binding motifs including both common transcription factors and nuclear hormone receptors within ME1 and ME2. Specifically, a functionally conserved liver X receptor (LXR) response element is present within both the enhancer sequences [14]. Indeed, pharmacological treatment with either TO901317, an agonist of LXR, or bexarotene, an agonist of retinoid X receptor (RXR), an obligate heterodimer of LXR, increases apoE secretion by astrocytes *in vitro* and in the brain *in vivo* [15]. LXR and RXR play a central role in maintaining lipid homoeostasis [16].

ApoE levels in the CNS have been shown to be dependent upon its lipidation by ABCA1, a member of the ATP-binding cassette family of active transporters [17]. ABCA1 expression is regulated by LXRs as demonstrated by an increase in ABCA1 expression both *in vitro* in astrocytes and *in vivo* in the brain following treatment with small molecule agonists of these nuclear receptors [18–23]. ABCA1 transfers cholesterol and phospholipids to apoE and leads to its secretion in high-density lipoprotein (HDL)-like particles by astrocytes. ABCA1 expression and activity is a critical regulator of apoE level and function in the brain. Deficiency of ABCA1 leads to poor lipidation and rapid degradation of brain apoE, and its overexpression leads to increased lipidation of apoE-containing HDL-like particles in the brain and cerebrospinal fluid (CSF) [17, 24].

While the roles of apoE in peripheral lipid transport and metabolism are well documented, its role in the CNS is less clear and it remains controversial whether increasing or decreasing brain apoE levels are beneficial [25, 26]. One argument is that the lipidation of apoE plays an important role in the beneficial functions of apoE and that changes in lipidation, such as those seen with apoE4, is what leads to the negative effects seen in apoE4 carriers [27–29]. The lipidation status is known to affect the function of apoE, such as its effect on the levels of Aβ in the brain [30]. Thus one can argue that increasing apoE lipidation will have a beneficial effect on apoE function in AD regardless of the apoE isoform [31, 32]. However there are reports showing gain of toxic effect of apoE4 in the brain, for example, apoE4 has been shown to increase tau deposition and tau-mediated neurodegeneration [33], and impairment of reelin signaling [34]. Nevertheless, given the evidence that apoE protein in the brain may be important for its normal function, particularly for neuronal and synaptic health and Aβ clearance, it is critical to understand the mechanisms regulating apoE expression and secretion in astrocytes, as well as regulation of its lipidating gene, ABCA1.

The regulation of apoE in the CNS, beyond control by nuclear receptors, is not well understood. With the goal to identify novel pathways regulating astrocytic apoE levels, a phenotypic screening strategy was employed utilizing libraries of annotated small molecules. 1,400 molecular mechanisms were tested, leading to the discovery that, in addition to LXR and RXR agonism, inhibition of histone deacetylases (HDACs) enhanced apoE secretion by astrocytes. Furthermore, siRNA mediated knockdown of the four classes of zinc-dependent HDACs indicated that pan class I HDAC inhibition alone was sufficient for enhancing astrocytic apoE secretion and for stimulating apoE and ABCA1 mRNA levels. Interestingly, unlike LXR and RXR agonism, the pan class I HDAC inhibition-mediated mechanism was not dependent on LXR activation. Treatment with MS275 and CI994, two potent pan class I HDAC inhibitors significantly increased apoE levels in human astrocytes. Additionally MS275 and CI994 elicited their effects in an LXR-independent manner. Furthermore a synergistic effect between MS275 and TO901317 was observed on their ability to increase apoE expression and secretion. These results demonstrated that pan class I HDAC inhibition is a novel mechanism for regulating astrocytic apoE expression and secretion.

## Materials and methods

### Cell culture and compound treatment

#### Human astrocytoma cells

The CCF-STTG1 cell line was obtained from ATCC and maintained in growth medium. Growth medium for CCF-STTG1 cells was a 2:1 ratio of high glucose DMEM to F12 (Life Technologies) supplemented with 10% FBS (Sigma), 2 mM Glutamax (Gibco) and 100 U/ml penicillin and streptomycin (Life Technologies). CCF-STTG1 cells have the apoE3/E4 genotype.

#### Human primary astrocytes

Primary human astrocytes (ScienCell Research Laboratories, 1800) were maintained in astrocyte medium (ScienCell Research Laboratories, 1801). Lot number 18709 has the apoE3/E3 genotype and lot number 06589 has the apoE3/E4 genotype and were used as indicated in the figure legends.

#### Compound treatment

Unless otherwise noted, TO901317 was obtained from SigmaAldrich (T2320), MS275 was obtained from Selleck Chemicals (S1053), and CI994 was obtained from SigmaAldrich (C0621) and stock solutions were prepared in dimethyl sulfoxide (DMSO). To evaluate the effect of compounds on mRNA expression or apoE secretion, CCF-STTG1 cells (40,000 cells/well) and primary human astrocytes (25,000 to 50,000 cells/well) were seeded in 96-well plates (Corning, 3596) in their respective growth medium. The following day, the growth medium was replaced with compounds diluted in serum-free culture medium. Dilutions were prepared such that the amount of DMSO was equal in all wells at 0.33%. Treatment duration lasted for 24 hours (RNA) or 48 hours (apoE secretion). For knock-down studies, CCF-STTG1 cells were seeded at 25,000 cells/well in 96-well plates and treated 48 hours later.

### Cell morphology

CCF-STTG1 cells and primary human astrocytes were plated (40,000 cells/well) on 96-well cell culture plates (Corning, 3596), treated with MS275, CI994 or DMSO for 48 hr and then transferred into the temperature and CO_2_ controlled (37°C, 5% CO_2_) environment of a Zeiss Celldiscoverer 7 microscope system. Live cell, phase gradient contrast images of individual field regions inside each well were automatically acquired using the ZEN Blue 2.3 software with a 5x/0.35 NA air objective and a pixel size of 0.908 μm on an Axiocam 506 camera. Representative figure images were selected with additional image postprocessing steps (contrast adjustment, field selection, and scalebar addition) performed in the Zeiss ZEN software (version 2.3) and ImageJ (version 1.51n).

### ApoE secretion

#### ApoE ELISA

For evaluating the effect of compounds on apoE secretion in screening format, CCF-STTG1 cells (10,000 cells/well) were seeded in 384-well plates in culture medium (3:1 High Glucose DMEM: F12, 10% HI FBS, Glutamax 10 mL/L, Pen-Strep 10 mL/L). The following day, the culture medium was replaced with 25 μl serum-free culture medium and compounds were added in 5 μl serum-free culture medium at a final concentration of 10 μM with 0.25% DMSO present. Treatment duration was 48 hours.

Secreted apoE from CCF-STTG1 cells was assessed using DELFIA technology (PerkinElmer). Briefly, capture-anti-apoE antibody (Millipore, AB947) was added at 25 μl per well (0.25 μg/ml) to 384-well plates (Nunc, 460518) which were incubated overnight at 4°C. After four washes with 80 μl of wash buffer (PBS, 0.05% Tween 20), wells were blocked with 50 μl of assay buffer (PBS, 0.05% Tween 20, 1% BSA (Sigma, A-7030)) for 2 hours at room temperature. The wells were then washed four times with 80 μl wash buffer, followed by addition of 7.5 μl assay buffer and 2.5 μl supernatant from the compound treated plates and allowed to incubate overnight at 4°C. The wells were then washed four times with 80 μl wash buffer, followed by the addition of 10 μl (0.24 μg/ml) of the anti-apoE detection antibody (Meridian, K74180B) in assay buffer and allowed to incubate for 2 hours at room temperature. The wells were then washed four times with 80 μl of wash buffer, followed by the addition of 10 μl (0.5 μg/ml) europium-streptavidin solution (Perkin Elmer, 1244–360) in assay buffer containing 20 μM EDTA and allowed to incubate for 20 minutes at room temperature. Plates were read on an Envision plate reader (PerkinElmer), with excitation at 320 nm and emission at 665 nm. The apoE standard curve was generated using isolated human apoE (Meridian, A50120H). The data were analyzed as percent effect compared to the positive control TO901317 (1 μM) set at 100% effect.

#### ApoE AlphaLISA

Secreted apoE from CCF-STTG1 cells and primary human astrocytes was assessed using AlphaLISA technology (PerkinElmer). Biotinylated anti-apoE antibody (1.2 nM, Meridian, K74180B) and purified anti-apoE antibody (Millipore, AB947) conjugated to AlphaLISA acceptor beads (40 μg/ml, PerkinElmer) were diluted in the assay buffer (30 mM Tris pH 7.5, 0.02% Tween-20, 0.02% Casein). Both the above reagents were added to a 384-well plate (5 μl/well) followed by addition of 5 μl of conditioned media collected from astrocytes. Following a one hour incubation at room temperature, 5 μl of streptavidin donor beads (PerkinElmer), diluted to 160 μg/ml in assay buffer, was added to each well and incubated in the dark for 30 minutes at room temperature. Plates were read on an Envision plate reader (PerkinElmer). ApoE standard curve was generated using isolated human apoE (Meridian, A50120H) as described above.

### siRNA knock-down

Cells were reverse transfected with Silencer Select siRNA oligonucleotides (Invitrogen) using Lipofectamine RNAiMAX reagent (Life Technologies). For CCF-STTG1 cells, Lipofectamine RNAiMAX (μl):siRNA (pmol) ratio was 20:1, with a final total siRNA concentration of 40 nM (6-well plate) or 48 nM (96-well plate). For primary astrocytes, Lipofectamine RNAiMAX (μl):siRNA (pmol) ratio was 3:1, with a final total siRNA concentration of 10 nM (96-well plate). When multiple siRNAs were co-transfected, equimolar amounts of each oligo were used, while maintaining the final concentration as mentioned above. Silencer Select Negative Control No. 1 or No. 2 (Invitrogen) or siGENOME Non-Targeting siRNA Pool #2 (Dharmacon) was used as a control. The siRNA oligonucleotides used are listed in S2 Table. 48 hours post-transfection, medium was changed to reduced-serum (2%) or serum-free medium with or without compound treatment for CCF-STTG1 cells and N2B27 for primary astrocytes and cells were harvested for RNA extraction at 72 hours and media was harvested at 96 hours post-transfection for apoE secretion. The lipid only condition refers to treatment with Lipofectamine RNAiMAX without any siRNA.

### RNA expression analysis

The method used for RNA isolation was dependent on the scale of the experiment. The TaqMan Cells-to-Cτ kit (Life Technologies) was used for RNA isolation and cDNA synthesis from cells in the 96-well plate format. For other formats, total RNA was extracted using RNeasy Mini Kits (Qiagen) and 1 μg of total RNA was used for reverse transcription using High Capacity RNA-to-cDNA Master Mix (Life Technologies) as per manufacturer’s instructions. For cDNA obtained from cells subjected to siRNA mediated knockdown, a pre-amplification step was performed using TaqMan PreAmp master mix (Life Technologies) prior to gene expression analysis. The cDNA or pre-amplification product was diluted 10-fold in TE buffer and used to evaluate expression of target genes by TaqMan real-time quantitative PCR using TaqMan Fast Advanced Master Mix (Applied Biosystems) and TaqMan gene expression assays (Applied Biosystems, S3 Table) in Applied Biosystems 7900HT, Applied Biosystems ViiA7 real-time PCR systems or the Bio-Rad CFX384 Real-Time PCR Detection System. Relative RNA quantity was calculated using the comparative C_T_ method using endogenous control genes for normalization.

### Binding assay

To determine binding of compounds to LXRs, compounds were shipped to Invitrogen for use in the Lanthascreen LXRα (PV4655) and LXRβ (PV4658) coactivator assays.

### Statistics

Data are represented as mean +/- standard error of the mean (SEM) of the depicted number of replicates or independent experiments. Statistical significance was assessed with GraphPad Prism 5 or 7 software, with one-way ANOVA analysis followed by Dunnett’s Multiple Comparison Test, unless otherwise noted.

## Results

### Identification of a novel enhancer of astrocytic apoE secretion from a chemical library screen

**Fig 1:**
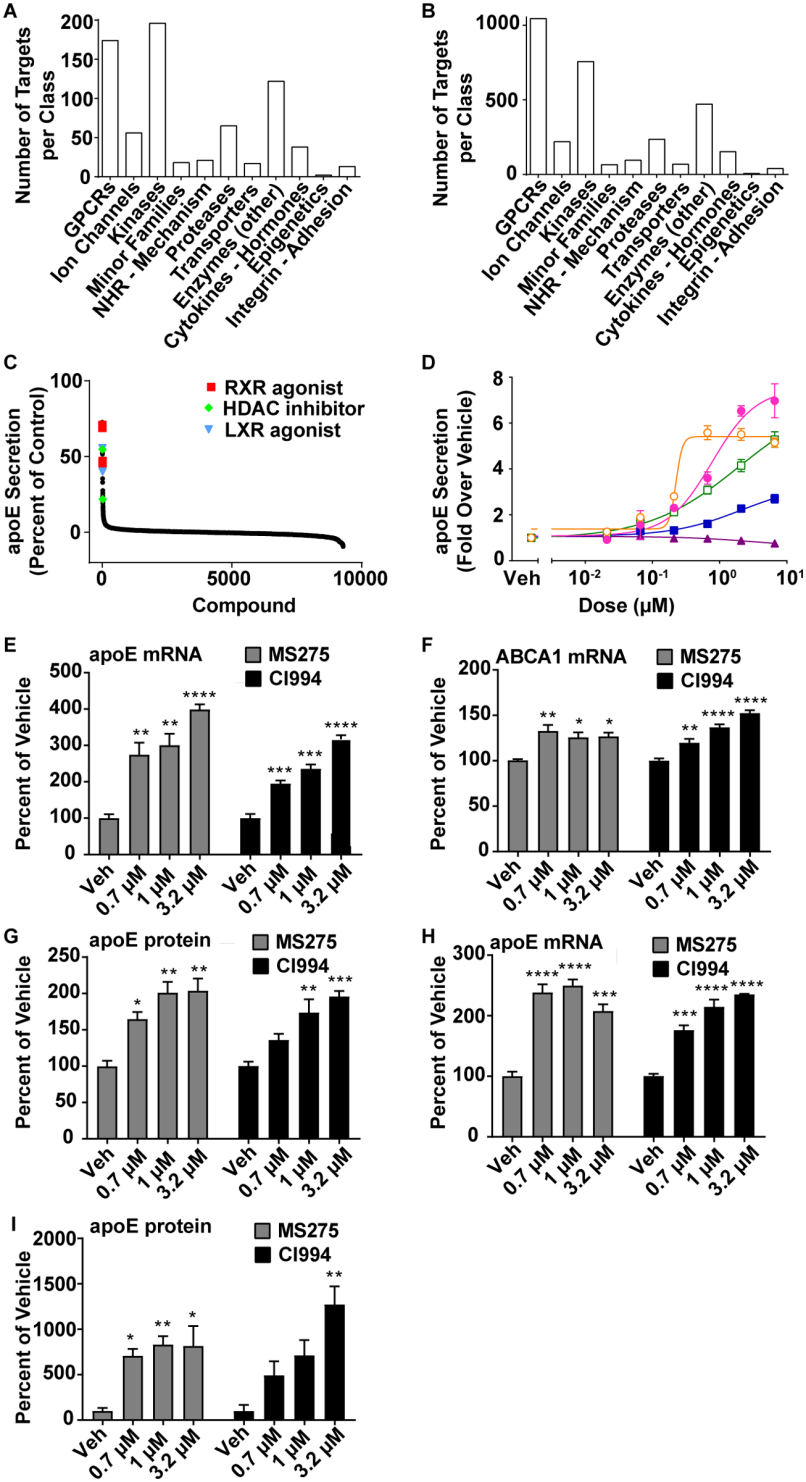
Pan class I HDAC inhibition increases astrocytic apoE levels. (*A*) The number of targets per class and (*B*) compounds per target class in Pfizer’s annotated chemogenomics library. (*C*) Number of library compounds resulting in apoE secretion from the CCF-STTG1 human astrocytoma cells following 10 μM treatment for 48 hours. ApoE levels were normalized to those that were obtained following exposure to 2 μM of TO901317. (*D*) Concentration-dependent increase in apoE secretion from CCF-STTG1 cells following treatment with HDAC inhibitors: MS275 (o), CI994 (●), SAHA (□) and BML-281(■) and TP38 (▲). Data are normalized to vehicle treated cells. (*E*) Concentration-dependent increase in apoE mRNA levels in CCF-STTG1 cells following exposure to MS275 and CI994. (*F*) Concentration-dependent increase in ABCA1 mRNA levels following exposure to MS275 and CI994. (See S3 Table for gene expression assays) (G) Concentration-dependent increase in apoE protein secretion from CCF-STTG1 cells following exposure to MS275 and CI994. (*H*) Concentration-dependent increase in apoE mRNA levels in primary human astrocytes following exposure to MS275 and CI994. (*I*) Concentration-dependent increase in apoE protein secretion in primary human astrocytes, expressing apoE3/E3 genotype (lot number 18709), following exposure to MS275 and CI994. Data were normalized to vehicle treated cells. Compounds for Fig 1A-D were synthesized at Pfizer. All data are represented as values ± SEM with n = 3, * p<0.05, ** p<0.01, *** *p*<0.001, **** p<0.0001.

To identify novel pathways and mechanisms involved in the regulation of apoE secretion, a phenotypic screening strategy was employed. The level of apoE secreted into media by a human astrocytoma cell line (CCF-STTG1) was measured following exposure to compounds from several annotated chemical libraries including a focused library from the Pfizer compound collection (chemogenomics library). The chemogenomics library consisted of 3,180 unique compounds covering 723 distinct biological mechanisms that were distributed into 11 gene classes (Fig 1A and 1B; S1 Text). We also used this approach to measure apoE secretion from CCF-STTG1 cells after exposure to compounds in three other libraries: a chemical library composed of past clinical candidates from the Pfizer compound collection, an epigenetics-focused library, and the LOPAC library (Sigma-Aldrich). From a total of 1,400 annotated mechanisms from across the libraries, we identified three classes of compounds that enhanced apoE secretion from CCF-STTG1 cells (Fig 1C). As expected both LXR and RXR/RAR (retinoic acid receptor) agonists were identified as potent mechanisms that resulted in robust secretion of apoE into the media. The amount of apoE secreted following exposure to 10 μM of the LXR and RXR/RAR compounds was about 40–70% of the amount secreted following treatment with TO901317 (2 μM), a pan LXR agonist [15, 22]. A third group of hits belonging to the pan class I HDAC inhibitor class of compounds was also identified. The two pan class I HDAC inhibitor hits identified were MS275 and CI994, which increased apoE secretion by about 22% and 55% respectively, compared to TO901317. These compounds had not been previously identified as pharmacological tools to increase apoE secretion. Based on these results, we concluded that pan class I HDAC inhibition could represent a novel mechanism for regulating astrocytic apoE secretion.

### Pan class I HDAC inhibitors increase astrocytic apoE secretion

To further explore the finding that pan class I HDAC inhibition results in increased astrocytic apoE secretion, a set of well-known HDAC inhibitors; MS275, SAHA, CI994, BML-281 and TP38, were characterized. These structurally differentiated HDAC inhibitors with varying degrees of HDAC sub-type selectivity were either purchased or synthesized according to published procedures [35–42]. The effect on apoE secretion from CCF-STTG1 cells following exposure to these structurally distinct HDAC inhibitors was then investigated (Fig 1D). A concentration dependent increase in astrocytic apoE secretion in response to treatment with MS275 (EC_50_: 0.22 μM), CI994 (EC_50_: 0.77 μM), SAHA (EC_50_: 2.0 μM), and BML-281 (EC_50_: 2.1 μM) was found. However, TP38, a class II selective HDAC inhibitor did not stimulate apoE secretion (Fig 1D).

Since HDACs are chromatin modifying enzymes, it was next investigated whether pan class I HDAC inhibitors directly altered apoE mRNA levels in CCF-STTG1 cells. Treatment of CCF-STTG1 cells with MS275 or CI994, the two most potent class I HDAC inhibitors identified in the phenotypic screen, resulted in a concentration-dependent increase in apoE mRNA levels (Fig 1E). Given that ABCA1 plays a key role in regulation of proper lipidation of apoE and its level in the CNS [17], mRNA levels of ABCA1 were also measured. Similar to apoE, the levels of ABCA1 mRNA increased following MS275 and CI994 exposure (Fig 1F). MS275 and CI994 treatment also resulted in a concentration-dependent increase in apoE secretion in CCF-STTG1 cells (Fig 1G). To confirm that the effect of pan class I HDAC inhibitors on apoE would occur in a native cell system, primary human astrocytes were also utilized. Following MS275 and CI994 treatment, a significant concentration-dependent enhancement in apoE mRNA expression (Fig 1H) and apoE protein secretion (Fig 1I) was observed in human primary astrocytes expressing apoE3/E3 genotype. Additionally, MS275 stimulated apoE secretion from primary human astrocytes with apoE3/E4 genotype (S1 Fig), indicating that the increase in apoE secretion was present in both apoE3/E3 and apoE3/E4 expressing astrocytes. Evaluation of cell health was done by assessing any changes in cell morphology by imaging. Neither cell death nor an effect on cell morphology was observed following treatment with either compound (S2 Fig). Taken together, these data demonstrated that pan class I HDAC inhibitors represent a novel compound class that stimulates apoE secretion by astrocytes.

### Pan class I HDAC knockdown is sufficient to increase apoE secretion from astrocytes

**Fig 2:**
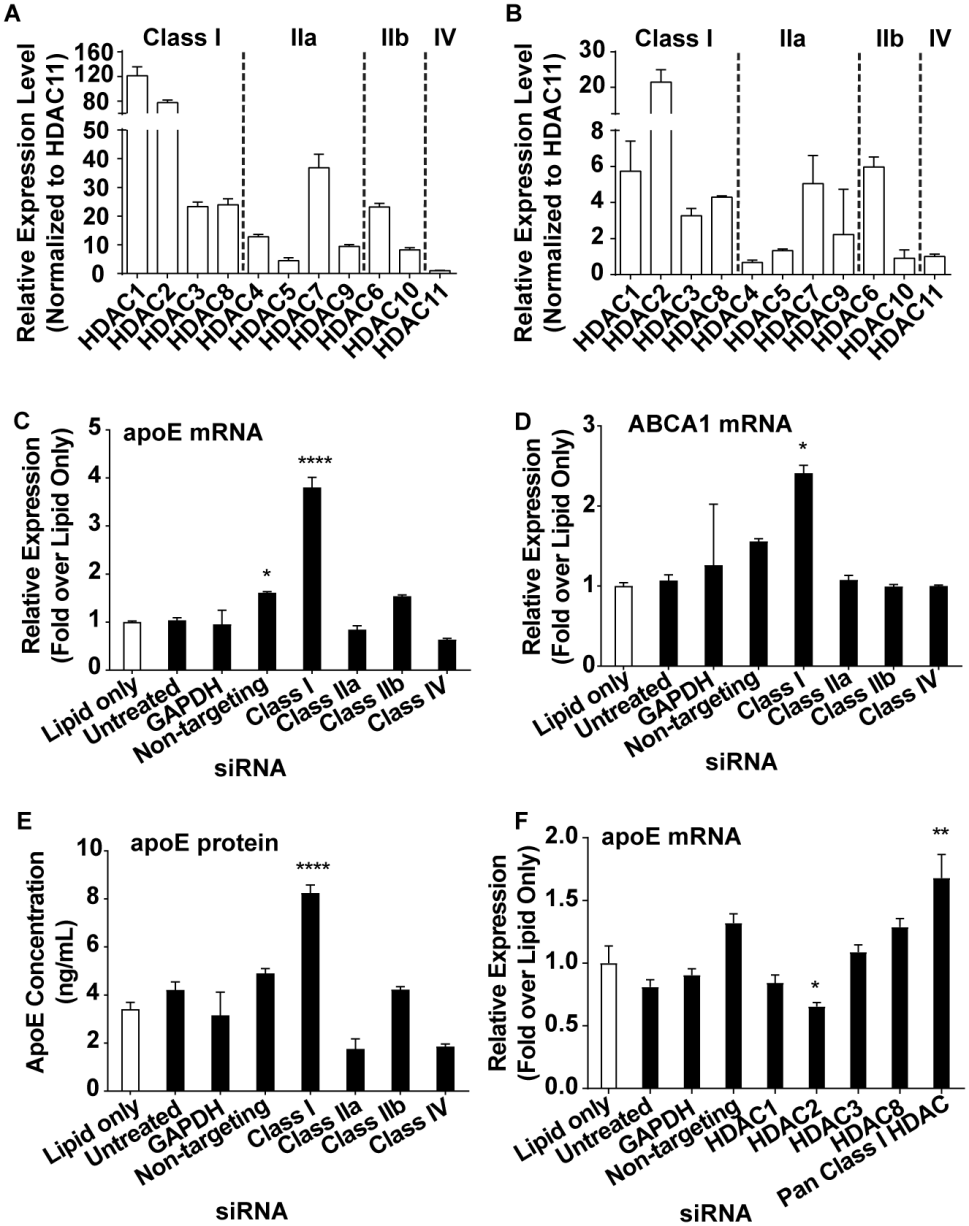
Pan class I HDAC knockdown increases apoE secretion by astrocytes. Relative mRNA expression of the zinc-dependent HDACs normalized to relative HDAC11 expression in total RNA isolated from CCF-STTG1 cells (*A*) and primary human astrocytes (*B*) (n = 4 per box). Effect of knocking down each of the four classes of zinc-dependent HDACs separately on relative apoE mRNA (*C*), relative ABCA1 mRNA (*D*) and secreted apoE protein levels (*E*) in CCF-STTG1 cells (n = 3 per condition). Relative apoE and ABCA1 mRNA levels obtained after knocking down HDACs were normalized to vehicle treated cells. (*F*) Effect of knocking down pan class I HDAC and each of the four class I HDAC members on relative apoE mRNA levels in CCF-STTG1 cells. Relative apoE mRNA levels obtained after knocking down HDACs were normalized to lipid only transfected cells. S3 and S4 Figs show siRNA knockdown selectivity and specificity for each member of the HDACs (see S2 and S3 Tables for siRNA oligonucleotides and gene expression assays, respectively). All data are represented as values ±SEM with n = 3; * *p*<0.05, **p<0.01, **** *p*<0.0001.

To further explore the nature of HDAC inhibition required for stimulation of apoE expression and secretion, each of the four zinc-dependent HDAC classes in CCF-STTG1 cells was knocked down using a pan HDAC siRNA approach. First, the relative mRNA expression for each of the eleven zinc-dependent HDAC members, in both CCF-STTG1 cells and primary human astrocytes, was determined (Fig 2A and 2B). Both cell systems expressed all members of the zinc-dependent HDACs with the general distribution pattern of expression of the various HDAC members being similar between the systems. For example, in both cell systems, HDAC1 and HDAC2, which are members of class I HDACs, showed higher expression relative to other members within the same class and other HDAC classes. In class II, HDAC6 and HDAC7 showed the highest expression relative to other members within that class. However, a much higher expression level, which ranged between four- and twenty-fold, was observed for different HDACs in CCF-STTG1 cells relative to their counterpart measured in primary human astrocytes. For example, HDAC1 had about twenty-fold and HDAC2 had about four-fold higher relative mRNA levels in the CCF-STTG1 cells than that in primary human astrocytes.

Each of the four classes of HDACs was knocked down and the effect on mRNA levels of apoE and ABCA1 and on apoE protein secretion in CCF-STTG1 cells was evaluated. Significant siRNA mediated knockdown was achieved for all four classes of HDACs (S3 Fig). However, a significant increase in apoE mRNA level was observed only upon knockdown of class I HDACs (Fig 2C). Specifically, pan class I HDAC knockdown led to about a 3.8 fold increase in apoE mRNA. Classes IIa, IIb, and IV HDAC knockdown did not significantly affect apoE mRNA levels. Furthermore, only pan class I HDAC knockdown led to a significant increase in ABCA1 mRNA expression, whereas knocking down HDAC classes IIa, IIb and IV did not significantly alter ABCA1 mRNA levels (Fig 2D). The increase in apoE mRNA expression resulting from knocking down class I HDACs was reflected in enhanced apoE secretion into the media (Fig 2E). No significant effect on apoE secretion was observed with classes IIa, IIb, or IV HDAC knockdown (Fig 2E). Collectively, these data indicated that pan class I HDAC inhibition was responsible for the increase in astrocytic apoE and ABCA1 mRNA levels and the enhanced apoE secretion.

Next it was investigated whether knocking down individual members of the class I HDAC family in CCF-STTG1 cells could increase apoE mRNA expression or if pan class I HDAC knockdown, which involved collective knockdown of all the four class I HDAC members (HDAC1, 2, 3 and 8), was required. While selective and significant reductions in the mRNA levels for each of the individual members of class I HDACs were observed (S4 Fig), a significant increase in apoE mRNA levels did not occur following knockdown of each individual member. However, when class I members were knocked down together (pan class I) a significant increase in apoE mRNA levels was observed (Fig 2F, S1 Table). Interestingly, HDAC2 knockdown led to a significant decrease in apoE mRNA. These data suggested that a synergistic interaction between two or more members of class I HDACs was responsible for the observed increase in apoE expression.

### Pan class I HDAC inhibition has a distinct molecular signature from pan LXR agonism

**Fig 3:**
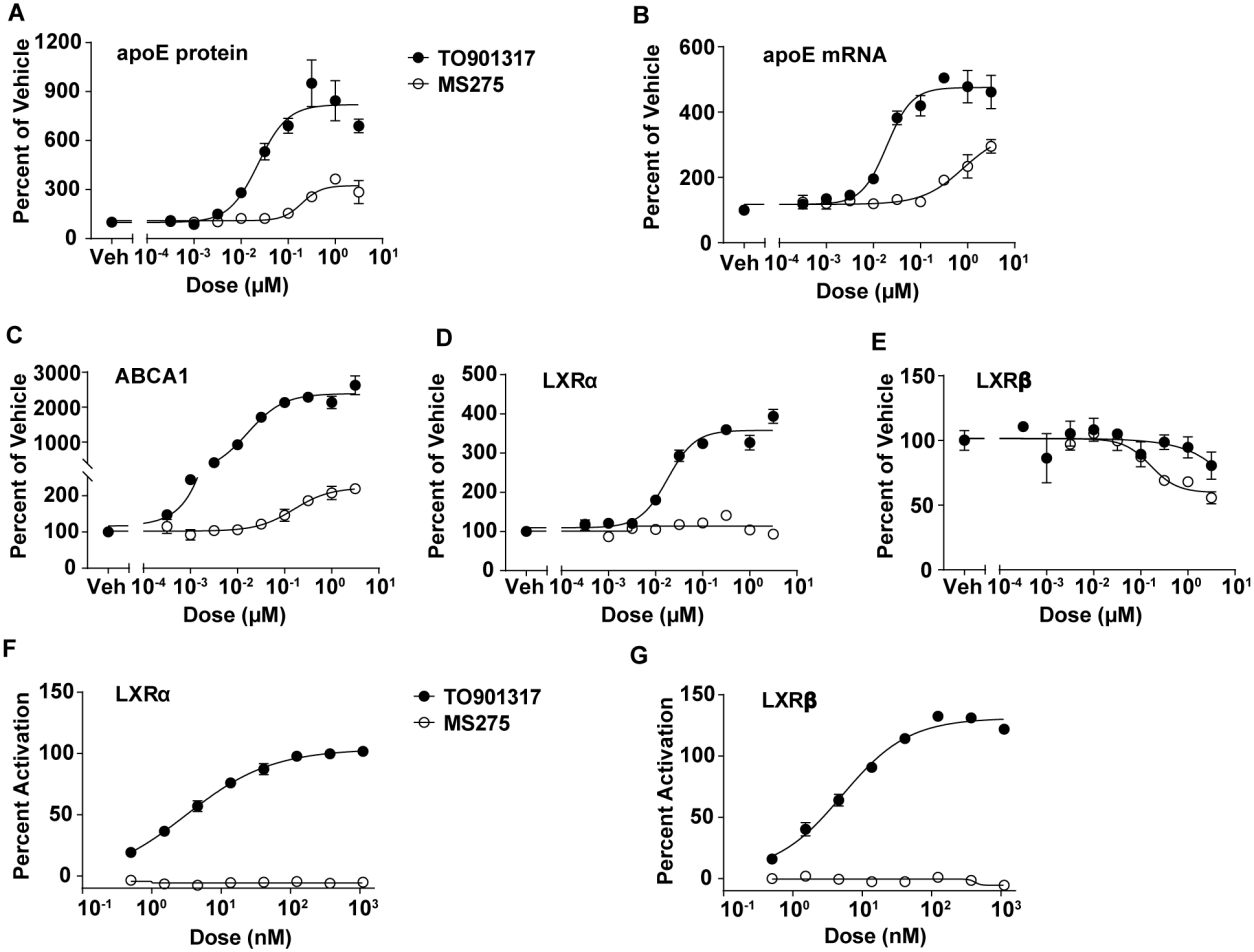
A pan class I HDAC inhibitor increases astrocytic apoE expression and secretion differently from a prototypic pan LXR agonist. Concentration-dependent effect of MS275 (o) and TO901317 (●), on secreted apoE protein (*A*) and relative levels of apoE mRNA (*B*), ABCA1 mRNA (*C*), LXRα mRNA (*D*) and LXRβ mRNA (*E*) in CCF-STTG1 cells. The amount of apoE protein secreted into the media and the relative mRNA levels of apoE, ABCA1, LXRα and LXRβ after compound treatment were normalized to those obtained from vehicle treated controls. The average of GAPDH and 18S mRNA were used as endogenous controls for mRNA normalization. See S3 Table for gene expression assays. TO901317 and MS275 were synthesized at Pfizer and their binding to LXRα (*F*) and LXRβ (*G*) was determined using the Lanthascreen assay (Life Technologies). All data are represented as values ±SEM.

LXR agonists, one of the three key classes of compounds identified from the phenotypic screen, have been reported previously to increase apoE expression and secretion [9, 15, 22]. To further understand if there was a mechanistic similarity between the pan class I HDAC inhibitors and LXR agonists, the effects of MS275 and TO901317 on astrocytic apoE expression were compared. TO901317 treatment led to a concentration-dependent increase in apoE protein secretion (EC_50_: 23 nM; Fig 3A) as well as apoE mRNA levels (EC_50_: 19 nM; Fig 3B). Though less potent than TO901317, MS275 also increased secreted apoE levels (EC_50_: 196 nM, Fig 3A) and apoE mRNA expression (EC_50_: 806 nM, Fig 3B). Because lipidation of apoE by ABCA1 is critical for its function, the regulation of ABCA1 by TO901317 and MS275 was also compared. Both TO901317 and MS275 increased ABCA1 mRNA levels (EC_50_: 16 nM and 156 nM respectively, Fig 3C), though the magnitude of MS275 mediated increase was much lower than that of TO901317. The effect of the two compounds on LXRα and LXRβ mRNA expression was compared as well. LXRα can induce its own gene expression following the binding of an agonist, whereas LXRβ has not been reported to induce its own expression in a similar auto-regulatory loop [43]. As expected, a concentration-dependent upregulation of LXRα mRNA (EC_50_: 18 nM) was observed after exposure to TO901317 (Fig 3D). Surprisingly, MS275 treatment did not alter LXRα mRNA levels (Fig 3D). LXRβ mRNA levels were not increased after exposure to either compound (Fig 3E). These results suggested that MS275 did not bind directly to LXR. To test whether differences in LXR binding could explain this finding, the ability of MS275 to bind to the ligand-binding domain of LXRα and LXRβ was compared with that of TO901317 using the Lanthascreen LXR coactivator assays (Invitrogen). TO901317 showed binding to both LXRα (EC_50_: 3.7 nM, Fig 3F) and LXRβ (EC_50_: 4.3 nM, Fig 3G). In contrast, MS275 did not demonstrate any binding to either LXR isoforms up to 1 μM (Fig 3F and 3G). Together, these data suggested that the MS275 elicited its effect on astrocytic apoE independent of direct LXR activation.

**Fig 4:**
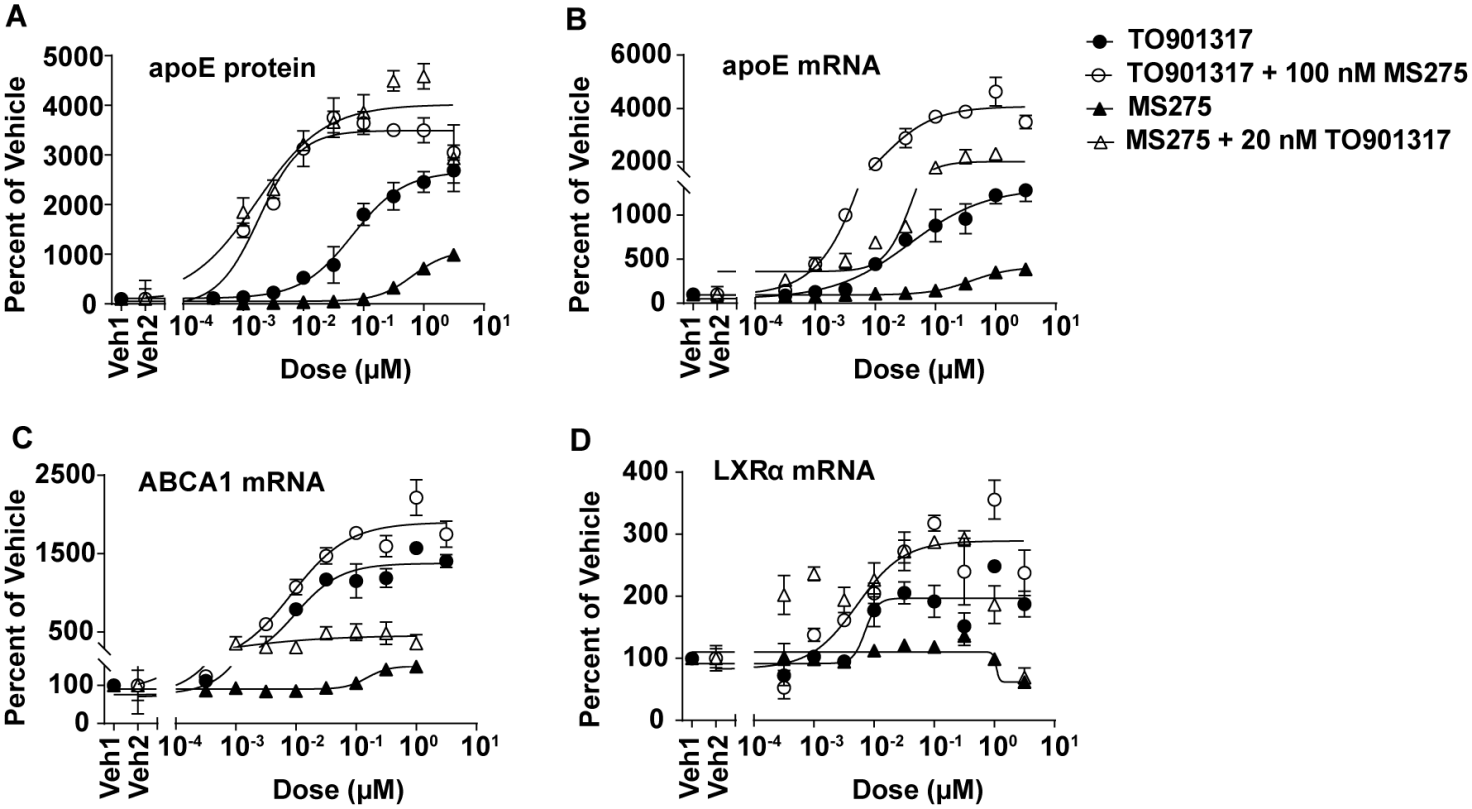
A pan class I HDAC inhibitor works synergistically with a pan LXR agonist on the expression of apoE but not ABCA1 or LXR. Treatment of TO901317 (●), MS275 (▲) or co-treatment of the compounds (TO901317 + 100 nM MS275 (o) or MS275 + 20 nM TO901317 (Δ)) on the levels of secreted apoE protein (*A*), and the levels of apoE mRNA (*B*), ABCA1 mRNA (*C*), and LXRα mRNA (*D*) in CCF-STTG1 cells. For co-treatment, the amount of apoE protein and apoE, ABCA1, and LXRα mRNA levels were normalized to vehicle treated controls (Veh1) and the effect of the constant compound (100 nM MS275 or 20 nM TO901317, denoted Veh2) was subtracted out after normalization. The average of GAPDH and 18S mRNA were used as endogenous controls for normalization of mRNA levels. See S3 Table for gene expression assays. All data are represented as values ±SEM.

After demonstrating that MS275 elevates apoE, as does a pan LXR agonist, it was next explored if the two mechanisms were mutually exclusive or whether they work synergistically to regulate apoE expression. Co-treatment studies were undertaken in which CCF-STTG1 cells were treated with a dose titration of TO901317 in the presence of 100 nM MS275 or a dose titration of MS275 in the presence of 20 nM of TO901317. These experiments were designed to determine how the two compounds affect the regulation of apoE levels in combination. Co-treatment of TO901317 and MS275 dramatically increased both apoE secretion (Fig 4A) and mRNA expression (Fig 4B) compared to the effect of either treatment alone, in a synergistic manner. Notably, co-treatment enhanced the potency for apoE secretion and apoE mRNA expression for both compounds, as evidenced by a leftward shift in their EC_50_ values. With TO901317 treatment alone the EC_50_ for apoE secretion was 6.6 nM and apoE mRNA was 37 nM, but with co-treatment of 100 nM MS275 the EC_50_ values shifted to 1.8 nM and 12 nM, respectively. With MS275 treatment alone the EC_50_ was 648 nM for apoE secretion and 386 nM for apoE mRNA, but with co-treatment of 20 nM TO901317, the EC_50_ values shifted to 1.7 nM and 42 nM, respectively. ABCA1, LXRα, and LXRβ mRNA levels were also evaluated. While both compounds individually increased ABCA1 mRNA (Fig 4C), the co-treatment conditions only showed a slight enhancement of expression, but no shift in the EC_50_ values. LXRα was only increased by TO901317 (Fig 4D) and LXRβ was unaffected by either compound individually or in combination (S5 Fig). Together these data suggested that a pan LXR agonist and pan class I HDAC inhibitor elicited their effects on apoE through separate but synergistic pathways.

### Pan class I HDAC inhibition-mediated effect is LXR-independent

**Fig 5:**
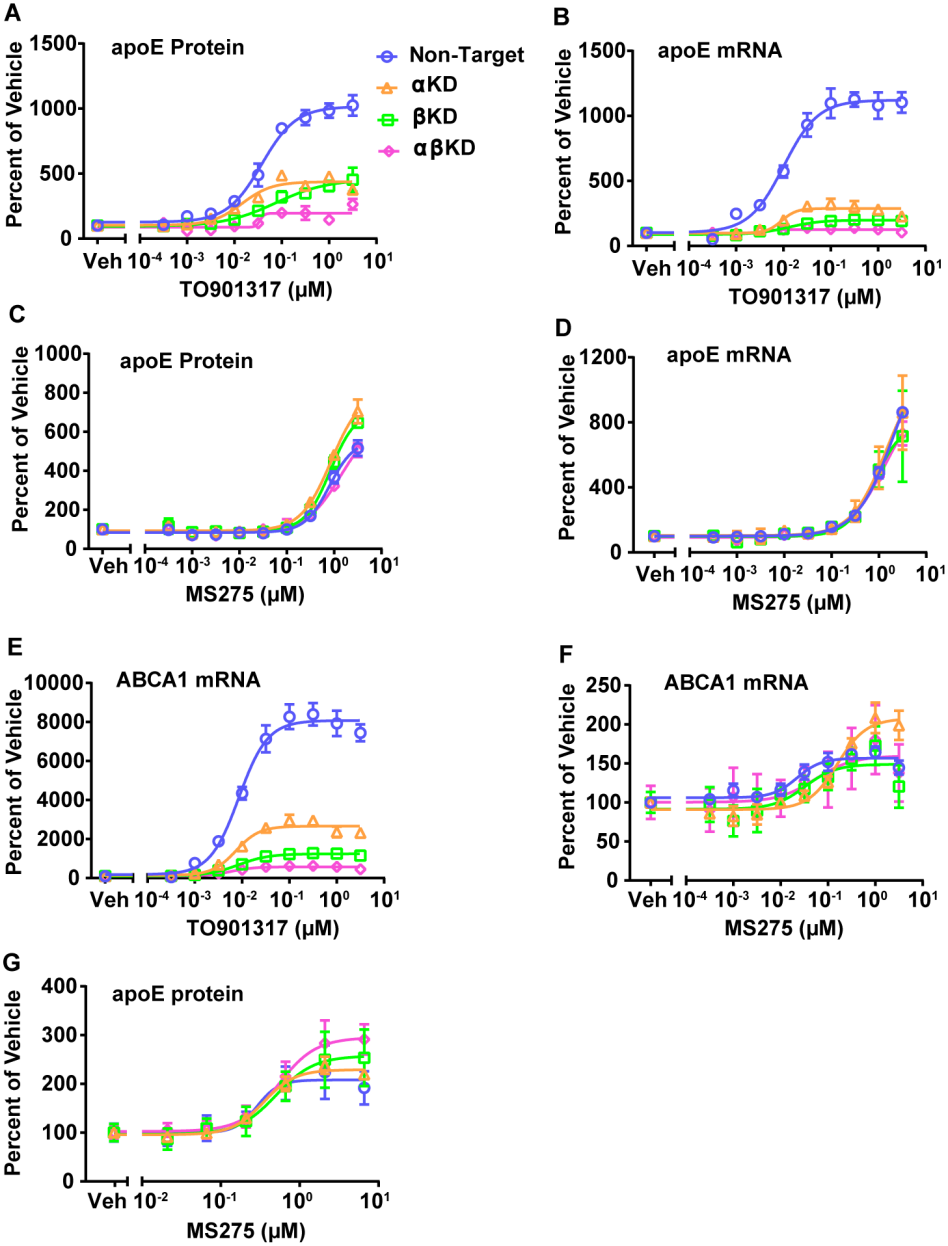
Pan class I HDAC inhibition increases astrocytic apoE secretion independent of LXR activation. Specific siRNA oligonucleotides were used to knock down LXRα (αKD) and LXRβ (βKD) either separately or in combination (αβKD) in CCF-STTG1 cells (see S2 Table for siRNA oligonucleotides). Concentration-dependent effect of exposure to TO901317 on apoE protein secreted into the media (*A*); and on apoE mRNA levels (*B*) upon knocking down LXRα and LXRβ separately or in combination. Concentration-dependent effect of exposure to MS275 on relative apoE protein secreted into the media (*C*); and on apoE mRNA levels (*D*) upon knocking down LXRα and LXRβ separately or in combination. Concentration-dependent effect of exposure to TO901317 (*E*) and MS275 (*F*) on relative ABCA1 mRNA level upon knocking down LXRα and LXRβ separately and in combination. (*G*) Concentration-dependent response of apoE protein secreted from primary human astrocytes (lot number 06589) after exposure to MS275 upon knocking down LXRα and LXRβ separately or in combination. For all, levels were normalized to the respective vehicle treated control. See S6 Fig for knock-down efficiencies of LXRα and LXRβ genes and S3 Table for gene expression assays. All data are represented as values ±SEM.

To determine if the pan class I HDAC inhibition results occurred via a direct or indirect effect on LXR, the LXRα and LXRβ genes were knocked down in CCF-STTG1 cells using targeted siRNA, separately and in combination (see S6A Fig for knockdown efficiency). The level of apoE mRNA and secretion of apoE protein were then measured after exposure to TO901317, MS275 or CI994. Following knockdown of LXRα or LXRβ separately and exposure to TO901317 there was a significant attenuation in the amount of apoE that was secreted into the media from CCF-STTG1 cells (Fig 5A). Similarly the level of apoE mRNA was significantly decreased when LXRα or LXRβ genes were knocked down (Fig 5B). When both LXRα and LXRβ were knocked down in combination, TO901317 exposure elicited no effect on either apoE secretion (Fig 5A) or apoE mRNA (Fig 5B). Knocking down either LXRα or LXRβ separately or in combination did not alter the stimulatory effect of MS275 on apoE secretion (Fig 5C) or apoE mRNA (Fig 5D). CI994 showed a similar response to knockdown of the two LXR isoforms on apoE mRNA expression and protein secretion (S7A and S7C Fig). Induction of ABCA1 mRNA by TO901317 showed a similar dependence on LXRs as to that demonstrated for apoE induction (Fig 5E). Stimulation of ABCA1 mRNA by MS275 and CI994 was also independent of LXRs (Fig 5F, S7B Fig). To strengthen the evidence that LXR activation was not required for apoE enhancement in response to MS275, the effect of knocking down LXRs on the ability of MS275 to enhance apoE protein secretion was evaluated in primary human astrocytes expressing apoE3/E4. Though significant and specific knockdown of the two LXRs was obtained (S6B Fig), neither single knockdown of LXRα or LXRβ, nor combined knockdown of the two LXR isoforms altered MS275 mediated stimulation of apoE secretion (Fig 5G). Taken together, these results demonstrated that the pan class I HDAC inhibition-mediated increase in astrocytic apoE occurred via an LXR-independent mechanism.

## Discussion

ApoE is an important brain lipid transport protein with critical roles in neuronal repair, synaptogenesis and clearance of amyloid plaques in the brain [2–4]. Additionally, apoE is essential for adult neurogenesis, maintenance of the neural progenitor pool in the adult dentate gyrus and is involved in synaptic pruning [44, 45]. In the present study, a phenotypic screening strategy was used to evaluate the Pfizer chemogenomics library and other annotated libraries to investigate the effect of 1,400 annotated mechanisms on apoE secretion from CCF-STTG1 cells. In addition to previously reported LXR and RXR agonists [15, 22], pan class I HDAC inhibitors were discovered to significantly increase apoE secretion (Fig 1C and 1D). Treatment with MS275 and CI994, the most potent pan class I HDAC inhibitors identified, enhanced apoE mRNA and ABCA1 mRNA levels and apoE secretion in CCF-STTG1 cells (Fig 1E–1G) and in primary human astrocytes (Fig 1H and 1I). Given that the stimulatory response was seen in astrocytes with both apoE3/E4 and apoE3/E3 genotypes (Fig 1E–1I and S1 Fig), collectively these data indicated that the pan class I HDAC inhibitor effect was independent of apoE3 or E4 genotypes. Furthermore, of the four classes of zinc-dependent HDACs, pan class I HDAC inhibition alone was sufficient for increasing apoE secretion and apoE and ABCA1 mRNA (Fig 2C–2F). The effect of pan class I HDAC inhibition was determined to be independent of LXR through knockdown of LXRα and LXRβ (Fig 5C, 5D, 5F and 5G). Based on the findings in this paper, pan class I HDAC inhibition is identified as a novel mechanism for regulating astrocytic apoE.

HDACs are a key class of chromatin modifying enzymes that alter histone-DNA interactions by removing acetyl groups from histones leading to gene silencing [46]. Histone acetylation mediated by increased activity of histone acetyl transferases (HATs) and decreased HDAC activity is important for regulating gene expression patterns involved in long-term memory formation [47–49]. Conversely, HDACs have been shown to be strong negative regulators of the processes leading to long-term memory. Though all zinc-dependent HDACs may interact with each other and other corepressors to form multi-protein complexes, specific members of the class I HDAC family have been reported to play a critical role in memory formation [48, 50–53]. HDAC2 has been shown to play an important role in neurodegenerative diseases, including AD, by inducing an epigenetic blockade on expression of genes involved in synaptic plasticity and cognition [54]. Additionally, HDAC1 and HDAC2 affect synapse development and neuronal function by forming a developmental switch that is dependent on the maturation state of neuronal networks [55]. Importantly, HDACs have also been demonstrated to play a key role in regulation of transcription factors and cofactors involved in regulation of genes in lipid metabolism [56]. The role of HDACs as an epigenetic regulator of cholesterol homeostasis is well known. Recruitment of class I and II HDACs to the gene promoter of cholesterol 7 α-hydroxylase (CYP7A1), a key cholesterol homeostasis enzyme, has been shown to repress CYP7A1 expression independent of farnesoid X receptor (FXR), a nuclear hormone receptor. Indeed, use of HDAC inhibitors such as valproic acid leads to an increase in CYP7A1 expression [57]. Collectively, these data suggest that HDACs play a central role in regulation of genes associated with lipid metabolism and their effect could in part be due to a nuclear hormone receptor independent mechanism.

Intriguingly, LXR activation was not required for the pan class I HDAC inhibition mediated increase in astrocytic apoE levels (Fig 5C, 5D and 5G). In contrast, TO901317 mediated enhancement of apoE required both LXRα and LXRβ (Fig 5A and 5B). Thus far, all known pathways regulating apoE expression have been shown to be dependent on nuclear hormone receptors such as LXR and RXR [15, 22, 23]. The current study provides an LXR-independent pathway for regulating astrocytic apoE expression and secretion. At the molecular level, HDACs have been shown to work in conjunction with corepressors, such as NCoRI, to modulate gene transcription [56, 57]. Therefore, it is plausible that pan class I HDAC inhibitors perturb the interaction between members of class I HDACs and corepressor(s), promoting the transcription of apoE without the involvement of LXRs. Further studies are needed to determine the factors and mechanism involved in apoE regulation by HDACs. The data illustrating the ability of pan class I HDAC inhibition to synergistically enhance the increased expression of apoE achieved by a pan LXR agonist (Fig 4), independent of an LXR-mediated mechanism (Fig 5) suggests a unique mechanism is involved.

In summary, using a novel phenotypic screening strategy, we have identified that pan class I HDAC inhibition represents a new mechanism for regulating astrocytic apoE expression and secretion. Given the critical role of apoE as a key lipid transporting protein in the brain, its link to neurodegenerative diseases such as AD and traumatic brain injury, emerging role in diseases such as multiple sclerosis [58] and the importance of class I HDACs in AD, regulation of astrocytic apoE via pan class I HDAC inhibition could provide a novel mechanism for therapeutic intervention for several debilitating CNS diseases.

## Supporting information

S1 Fig ApoE protein secretion from primary human astrocytes with apoE3/E4 genotype. ApoE protein secretion from CCF-STTG1 cells and primary human astrocytes (lot number 06589) expressing apoE3/E4 genotype shows a concentration-dependent increase following treatment with MS275. Data were normalized to vehicle treated cells. All data are represented as values ± SEM with **** p<0.0001.
(TIF)

S2 Fig Cell morphology following treatment with MS275 and CI994. CCF-STTG1 cells and primary human astrocytes were imaged live 48hr post treatment with MS275, CI994, or vehicle. (*A*) CCF-STTG1 cells treated with vehicle (left), 3.2 μM MS275 (middle) and 3.2 μM CI994 (right). (*B*) Primary human astrocytes treated with vehicle (left), 3.2 μM MS275 (middle) and 3.2 μM CI994 (right). Scale bar = 500 μm.
(TIF)

S3 Fig Knock-down efficiency and specificity for the four main classes of HDACs. Each of the four classes of HDACs were knocked down using siRNA oligonucleotides in CCF-STTG1 cells and effect of knockdown on relative mRNA levels of each HDAC member was evaluated. The percent knockdown was calculated by normalizing the relative HDAC expression upon knockdown to the “lipid only” control. (A) Effect of knockdown on class I HDAC members. Significant reduction in the expression of class I HDACs (HDAC 1, 2, 3 and 8) was observed in response to pan class I HDAC knockdown. (B) Effect on class IIa HDAC members. Class IIa HDAC knockdown led to a significant decrease in HDAC 5 and 7 expression, but it had no effect on expression of HDAC 4 and 9. Interestingly, knocking down class I HDACs also decreased expression levels of HDAC 7 and 9. (C) Effect on class IIb HDAC members. Class IIb HDAC knockdown led to a decrease in expression of HDAC 6 and 10. (D) Effect on HDAC11, class IV HDAC member. Interestingly, a cross-over effect of knocking-down one class of HDAC on members belonging to the classes IIa, IIb and IV was also observed (B, C and D) suggesting interactions between the expression of various HDAC members. See S2 Table for siRNA oligonucleotides and S3 Table for gene expression assays. All data are represented as values ± SEM (n = 3); * *p*<0.05, ** *p*<0.01; *** *p*<0.001, student’s t test.
(TIF)

S4 Fig Knock-down efficiency for class I HDAC members. Each of the four members of pan class I HDAC were knocked down using a 40 nM concentration of pan class I HDAC siRNA or specific class I HDAC oligonucleotide in CCF-STTG1 cells grown in 6-well format (n = 3 per condition). Medium was replaced 24 hours post-transfection to serum free medium. Total RNA was extracted from cells 72 hours post-transfection and gene expression was evaluated using reverse transcription followed by quantitative real-time PCR. RNA expression was normalized to RPL13 mRNA expression. The percent knockdown was calculated by comparing with “lipid only” control. Knocking down using either an siRNA oligonucleotide targeting a specific member or using pan class I HDAC siRNA led to a significant decrease in the four members belonging to class I HDAC and is shown as follows: (A) HDAC1; (B) HDAC2; (C) HDAC3; and (D) HDAC8. Interestingly, knocking down HDAC3 led to a slight decrease in HDAC1 mRNA as shown in (A). See S2 Table for siRNA oligonucleotides and S3 Table for gene expression assays. All data are represented as values ± SEM with effect significance designated as follows: *** *p*<0.001.
(TIF)

S5 Fig LXRβ mRNA following TO901317 and MS275 co-treatment. Treatment of TO901317 (●), MS275 (▲) or co-treatment of the compounds (TO901317 + 100 nM MS275 (o) or MS275 + 20 nM TO901317 (Δ)) on the levels of LXRβ mRNA. mRNA levels were normalized to vehicle treated controls (Veh1) and the effect of the constant compound (100 nM MS275 or 20 nM TO901317, denoted Veh2) was subtracted out after normalization. The average of GAPDH and 18S mRNA were used as endogenous controls for normalization of mRNA levels. See S3 Table for gene expression assays. All data are represented as values ±SEM.
(TIF)

S6 Fig Knock-down efficiency for LXRα and LXRβ. CCF-STTG1 cells (25,600 cells/well) or primary human astrocytes (22,500 cells/well) were grown in 96-well format and LXRα and LXRβ genes were knocked down either alone (LXRα KD or LXRβ KD) or in combination (LXRαβ KD) (n = 3 per condition). Total RNA was extracted and mRNA expression of LXRα and LXRβ genes were measured using quantitative real-time PCR and normalized to the average of GAPDH and 18s mRNA expression. See S2 Table for siRNA oligonucleotides and S3 Table for gene expression assays. (A) Significant knockdown (>55%) for LXRα and LXRβ genes was obtained when either was knocked down separately or in combination in CCF-STTG1 cells. (B) In primary human astrocytes (lot number 06589) greater than 60% knockdown was observed for LXRα and LXRβ genes both when knocked down separately or in combination. All data are represented as values ± SEM with effect significance designated as follows: * p<0.05, ****p <0.0001.
(TIF)

S7 Fig Effect of CI994 treatment on apoE and ABCA1 following LXR knockdown. CCF-STTG1 cells were treated with the pan class I HDAC inhibitor, CI994, following knockdown of LXRα and LXRβ. (*A*) Concentration dependent effect on apoE mRNA levels following exposure with CI994 upon knocking down LXRα and LXRβ, separately or in combination. (*B*) Concentration dependent effect on ABCA1 mRNA levels following exposure with CI994 upon knocking down LXRα and LXRβ, separately or in combination. (*C*) Concentration dependent effect on apoE protein levels secreted into the media following exposure with CI994 upon knocking down LXRα and LXRβ, separately or in combination. See S2 Table for siRNA oligonucleotides and S3 Table for gene expression assays. All data are represented as values ± SEM.
(TIF)

S1 Table Knock-down efficiency for pan class I HDACs. (DOCX)

S2 Table siRNA oligos used in knock-down experiments. (DOCX)

S3 Table Gene expression assays for real time PCR. (DOCX)

S1 Text Pfizer chemogenomics library. (DOCX)

## References

[1] Huang Y, Weisgraber KH, Mucke L, Mahley RW. Apolipoprotein E: diversity of cellular origins, structural and biophysical properties, and effects in Alzheimer’s disease. J Mol Neurosci. 2004;23(3):189–204. doi: 10.1385/JMN:23:3:189 15181247 10.1385/JMN:23:3:189

[2] Hauser PS, Narayanaswami V, Ryan RO. Apolipoprotein E: From lipid transport to neurobiology. Prog Lipid Res. 2011;50(1):62–74. doi: 10.1016/j.plipres.2010.09.001 20854843 10.1016/j.plipres.2010.09.001 PMC3022415

[3] Weisgraber KH, Mahley RW. Human apolipoprotein E: the Alzheimer’s disease connection. FASEB J. 1996;10(13):1485–94. 8940294 10.1096/fasebj.10.13.8940294

[4] Hauser PS, Ryan RO. Impact of apolipoprotein E on Alzheimer’s disease. Curr Alzheimer Res. 2013;10(8):809–17. 23919769 10.2174/15672050113109990156 PMC3995977

[5] Hatters DM, Peters-Libeu CA, Weisgraber KH. Apolipoprotein E structure: insights into function. Trends Biochem Sci. 2006;31(8):445–54. doi: 10.1016/j.tibs.2006.06.008 16820298 10.1016/j.tibs.2006.06.008

[6] Corder EH, Saunders AM, Strittmatter WJ, Schmechel DE, Gaskell PC, Rimmler JB, et al Apolipoprotein E, survival in Alzheimer’s disease patients, and the competing risks of death and Alzheimer’s disease. Neurology. 1995;45(7):1323–8. 7617191 10.1212/wnl.45.7.1323

[7] Farrer LA, Cupples L, Haines JL, Hyman B, Kukull WA, Mayeux R, et al Effects of age, sex, and ethnicity on the association between apolipoprotein e genotype and alzheimer disease: A meta-analysis. JAMA. 1997;278(16):1349–56. 9343467

[8] Ramaswamy G, Xu Q, Huang Y, Weisgraber KH. Effect of domain interaction on apolipoprotein E levels in mouse brain. J Neurosci. 2005;25(46):10658–63. doi: 10.1523/JNEUROSCI.1922-05.2005 16291938 10.1523/JNEUROSCI.1922-05.2005 PMC6725862

[9] Riddell DR, Zhou H, Atchison K, Warwick HK, Atkinson PJ, Jefferson J, et al Impact of apolipoprotein E (ApoE) polymorphism on brain ApoE levels. J Neurosci. 2008;28(45):11445–53. doi: 10.1523/JNEUROSCI.1972-08.2008 18987181 10.1523/JNEUROSCI.1972-08.2008 PMC6671315

[10] Sullivan PM, Han B, Liu F, Mace BE, Ervin JF, Wu S, et al Reduced levels of human apoE4 protein in an animal model of cognitive impairment. Neurobiol Aging. 2011;32(5):791–801. doi: 10.1016/j.neurobiolaging.2009.05.011 19577821 10.1016/j.neurobiolaging.2009.05.011

[11] Myklebost O, Rogne S. A physical map of the apolipoprotein gene cluster on human chromosome 19. Hum Genet. 1988;78(3):244–7. 2894348 10.1007/BF00291670

[12] Grehan S, Tse E, Taylor JM. Two distal downstream enhancers direct expression of the human apolipoprotein E gene to astrocytes in the brain. J Neurosci. 2001;21(3):812–22. 11157067 10.1523/JNEUROSCI.21-03-00812.2001 PMC6762321

[13] Shih S-J, Allan C, Grehan S, Tse E, Moran C, Taylor JM. Duplicated downstream enhancers control expression of the human apolipoprotein E gene in macrophages and adipose tissue. J Biol Chem. 2000;275(41):31567–72. doi: 10.1074/jbc.M005468200 10893248 10.1074/jbc.M005468200

[14] Laffitte BA, Repa JJ, Joseph SB, Wilpitz DC, Kast HR, Mangelsdorf DJ, et al LXRs control lipid-inducible expression of the apolipoprotein E gene in macrophages and adipocytes. Proc Natl Acad Sci U S A. 2001;98(2):507–12. doi: 10.1073/pnas.98.2.507 11149950 10.1073/pnas.021488798 PMC14617

[15] Liang Y, Lin S, Beyer TP, Zhang Y, Wu X, Bales KR, et al A liver X receptor and retinoid X receptor heterodimer mediates apolipoprotein E expression, secretion and cholesterol homeostasis in astrocytes. J Neurochem. 2004;88(3):623–34. 14720212 10.1111/j.1471-4159.2004.02183.x

[16] Hong C, Tontonoz P. Liver X receptors in lipid metabolism: opportunities for drug discovery. Nat Rev Drug Discov. 2014;13(6):433–44. doi: 10.1038/nrd4280 24833295 10.1038/nrd4280

[17] Wahrle SE, Jiang H, Parsadanian M, Legleiter J, Han X, Fryer JD, et al ABCA1 is required for normal central nervous system ApoE levels and for lipidation of astrocyte-secreted apoE. J Biol Chem. 2004;279(39):40987–93. doi: 10.1074/jbc.M407963200 15269217 10.1074/jbc.M407963200

[18] Costet P, Luo Y, Wang N, Tall AR. Sterol-dependent transactivation of the ABC1 promoter by the Liver X Receptor/Retinoid X Receptor. J Biol Chem. 2000;275(36):28240–5. doi: 10.1074/jbc.M003337200 10858438 10.1074/jbc.M003337200

[19] Riddell DR, Zhou H, Comery TA, Kouranova E, Lo CF, Warwick HK, et al The LXR agonist TO901317 selectively lowers hippocampal Aβ42 and improves memory in the Tg2576 mouse model of Alzheimer’s disease. Mol Cell Neurosci. 2007;34(4):621–8. doi: 10.1016/j.mcn.2007.01.011 17336088 10.1016/j.mcn.2007.01.011

[20] Lefterov I, Bookout A, Wang Z, Staufenbiel M, Mangelsdorf D, Koldamova R. Expression profiling in APP23 mouse brain: inhibition of Aβ amyloidosis and inflammation in response to LXR agonist treatment. Molecular Neurodegeneration. 2007;2(1):20. 17953774 10.1186/1750-1326-2-20 PMC2214725

[21] Terwel D, Steffensen KR, Verghese PB, Kummer MP, Gustafsson J-Å, Holtzman DM, et al Critical role of astroglial apolipoprotein E and liver X receptor-α expression for microglial Aβ phagocytosis. J Neurosci. 2011;31(19):7049–59. doi: 10.1523/JNEUROSCI.6546-10.2011 21562267 10.1523/JNEUROSCI.6546-10.2011 PMC6703224

[22] Cramer PE, Cirrito JR, Wesson DW, Daniel Lee CY, Karlo JC, Zinn AE, et al ApoE-directed therapeutics rapidly clear β-amyloid and reverse deficits in AD mouse models. Science. 2012;335(6075):1503–6. doi: 10.1126/science.1217697 22323736 10.1126/science.1217697 PMC3651582

[23] Mandrekar-Colucci S, Karlo JC, Landreth GE. Mechanisms underlying the rapid peroxisome proliferator-activated receptor-γ-mediated amyloid clearance and reversal of cognitive deficits in a murine model of Alzheimer’s disease. J Neurosci. 2012;32(30):10117–28. doi: 10.1523/JNEUROSCI.5268-11.2012 22836247 10.1523/JNEUROSCI.5268-11.2012 PMC3433721

[24] Hirsch-Reinshagen V, Zhou S, Burgess BL, Bernier L, McIsaac SA, Chan JY, et al Deficiency of ABCA1 Impairs Apolipoprotein E Metabolism in Brain. J Biol Chem. 2004;279(39):41197–207. doi: 10.1074/jbc.M407962200 15269218 10.1074/jbc.M407962200

[25] Kim J, Basak JM, Holtzman DM. The role of apolipoprotein E in Alzheimer’s disease. Neuron. 2009;63(3):287–303. doi: 10.1016/j.neuron.2009.06.026 19679070 10.1016/j.neuron.2009.06.026 PMC3044446

[26] Liu C-C, Kanekiyo T, Xu H, Bu G. Apolipoprotein E and Alzheimer disease: risk, mechanisms, and therapy. Nat Rev Neurol. 2013;9(2):106–18. doi: 10.1038/nrneurol.2012.263 23296339 10.1038/nrneurol.2012.263 PMC3726719

[27] LaDu MJ, Falduto MT, Manelli AM, Reardon CA, Getz GS, Frail DE. Isoform-specific binding of apolipoprotein E to beta-amyloid. J Biol Chem. 1994;269(38):23403–6. 8089103

[28] LaDu MJ, Pederson TM, Frail DE, Reardon CA, Getz GS, Falduto MT. Purification of apolipoprotein E attenuates isoform-specific binding to β-Amyloid. J Biol Chem. 1995;270(16):9039–42. 7721816 10.1074/jbc.270.16.9039

[29] Tokuda T, Calero M, Matsubara E, Vidal R, Kumar A, Permanne B, et al Lipidation of apolipoprotein E influences its isoform-specific interaction with Alzheimer’s amyloid beta peptides. Biochem J. 2000;348(Pt 2):359–65. 10816430 PMC1221074

[30] Jiang Q, Lee CYD, Mandrekar S, Wilkinson B, Cramer P, Zelcer N, et al ApoE promotes the proteolytic degradation of Aβ. Neuron. 2008;58(5):681–93. doi: 10.1016/j.neuron.2008.04.010 18549781 10.1016/j.neuron.2008.04.010 PMC2493297

[31] Boehm-Cagan A, Michaelson DM. Reversal of apoE4-driven brain pathology and behavioral deficits by bexarotene. J Neurosci. 2014;34(21):7293–301. doi: 10.1523/JNEUROSCI.5198-13.2014 24849361 10.1523/JNEUROSCI.5198-13.2014 PMC6608187

[32] Michaelson DM. APOE ε4: The most prevalent yet understudied risk factor for Alzheimer’s disease. Alzheimers Dement. 2014;10(6):861–8. doi: 10.1016/j.jalz.2014.06.015 25217293 10.1016/j.jalz.2014.06.015

[33] Shi Y, Yamada K, Liddelow SA, Smith ST, Zhao L, Luo W, et al ApoE4 markedly exacerbates tau-mediated neurodegeneration in a mouse model of tauopathy. Nature. 2017;549(7673):523–7. doi: 10.1038/nature24016 28959956 10.1038/nature24016 PMC5641217

[34] Chen Y, Durakoglugil MS, Xian X, Herz J. ApoE4 reduces glutamate receptor function and synaptic plasticity by selectively impairing ApoE receptor recycling. Proc Natl Acad Sci U S A. 2010;107(26):12011–6. doi: 10.1073/pnas.0914984107 20547867 10.1073/pnas.0914984107 PMC2900641

[35] Saito A, Yamashita T, Mariko Y, Nosaka Y, Tsuchiya K, Ando T, et al A synthetic inhibitor of histone deacetylase, MS-27-275, with marked in vivo antitumor activity against human tumors. Proc Natl Acad Sci U S A. 1999;96(8):4592–7. 10200307 10.1073/pnas.96.8.4592 PMC16377

[36] Suzuki T, Ando T, Tsuchiya K, Fukazawa N, Saito A, Mariko Y, et al Synthesis and histone deacetylase inhibitory activity of new benzamide derivatives. J Med Chem. 1999;42(15):3001–3. doi: 10.1021/jm980565u 10425110 10.1021/jm980565u

[37] Kraker AJ, Mizzen CA, Hartl BG, Miin J, Allis CD, Merriman RL. Modulation of histone acetylation by [4-(Acetylamino)-N-(2-Amino-phenyl) Benzamide] in HCT-8 colon carcinoma. Mol Cancer Ther. 2003;2(4):401–8. 12700284

[38] El-Beltagi HM, Martens ACM, Lelieveld P, Haroun EA, Hagenbeek A. Acetyldinaline: a new oral cytostatic drug with impressive differential activity against leukemic cells and normal stem cells—preclinical studies in a relevant rat model for human acute myelocytic leukemia. Cancer Res. 1993;53(13):3008–14. 8319208

[39] Stowell JC, Huot RI, Van Voast L. The Synthesis of N-Hydroxy-N'-phenyloctanediamide and Its Inhibitory Effect on Proliferation of AXC Rat Prostate Cancer Cells. J Med Chem. 1995;38(8):1411–3. 7731025 10.1021/jm00008a020

[40] Butler LM, Agus DB, Scher HI, Higgins B, Rose A, Cordon-Cardo C, et al Suberoylanilide hydroxamic acid, an inhibitor of histone deacetylase, suppresses the growth of prostate cancer cells in vitro and in vivo. Cancer Res. 2000;60(18):5165–70. 11016644

[41] Kozikowski AP, Tapadar S, Luchini DN, Kim KH, Billadeau DD. Use of the nitrile oxide cycloaddition (NOC) reaction for molecular probe generation: a new class of enzyme selective histone deacetylase inhibitors (HDACIs) showing picomolar activity at HDAC6. J Med Chem. 2008;51(15):4370–3. doi: 10.1021/jm8002894 18642892 10.1021/jm8002894 PMC3913184

[42] Baloglu E, Ghosh S, Lobera M, Schmidt D, inventors. Compounds and methods. Patent WO/2011/088192. 2013.

[43] Li Y, Bolten C, Bhat BG, Woodring-Dietz J, Li S, Prayaga SK, et al Induction of human liver X receptor α gene expression via an autoregulatory loop mechanism. Mol Endocrinol. 2002;16(3):506–14. doi: 10.1210/mend.16.3.0789 11875109 10.1210/mend.16.3.0789

[44] Yang C-P, Gilley JA, Zhang G, Kernie SG. ApoE is required for maintenance of the dentate gyrus neural progenitor pool. Development. 2011;138(20):4351–62. doi: 10.1242/dev.065540 21880781 10.1242/dev.065540 PMC3177307

[45] Chung W-S, Verghese PB, Chakraborty C, Joung J, Hyman BT, Ulrich JD, et al Novel allele-dependent role for APOE in controlling the rate of synapse pruning by astrocytes. Proc Natl Acad Sci U S A. 2016;113(36):10186–91. doi: 10.1073/pnas.1609896113 27559087 10.1073/pnas.1609896113 PMC5018780

[46] Chuang D-M, Leng Y, Marinova Z, Kim H-J, Chiu C-T. Multiple roles of HDAC inhibition in neurodegenerative conditions. Trends Neurosci. 2009;32(11):591–601. doi: 10.1016/j.tins.2009.06.002 19775759 10.1016/j.tins.2009.06.002 PMC2771446

[47] Swank MW, Sweatt JD. Increased histone acetyltransferase and lysine acetyltransferase activity and biphasic activation of the ERK/RSK cascade in insular cortex during novel taste learning. J Neurosci. 2001;21(10):3383–91. 11331368 10.1523/JNEUROSCI.21-10-03383.2001 PMC6762472

[48] Stefanko DP, Barrett RM, Ly AR, Reolon GK, Wood MA. Modulation of long-term memory for object recognition via HDAC inhibition. Proc Natl Acad Sci U S A. 2009;106(23):9447–52. doi: 10.1073/pnas.0903964106 19470462 10.1073/pnas.0903964106 PMC2695069

[49] Volmar C-H, Wahlestedt C. Histone deacetylases (HDACs) and brain function. Neuroepigenetics. 2015;1(Supplement C):20–7.

[50] Guan J-S, Haggarty SJ, Giacometti E, Dannenberg J-H, Joseph N, Gao J, et al HDAC2 negatively regulates memory formation and synaptic plasticity. Nature. 2009;459(7243):55–60. doi: 10.1038/nature07925 19424149 10.1038/nature07925 PMC3498958

[51] McQuown SC, Barrett RM, Matheos DP, Post RJ, Rogge GA, Alenghat T, et al HDAC3 is a critical negative regulator of long-term memory formation. J Neurosci. 2011;31(2):764–74. doi: 10.1523/JNEUROSCI.5052-10.2011 21228185 10.1523/JNEUROSCI.5052-10.2011 PMC3160172

[52] McQuown SC, Wood MA. HDAC3 and the molecular brake pad hypothesis. Neurobiol Learn Mem. 2011;96(1):27–34. 21521655 10.1016/j.nlm.2011.04.005 PMC3111848

[53] Bardai FH, Price V, Zaayman M, Wang L, D’Mello SR. Histone deacetylase-1 (HDAC1) Is a molecular switch between neuronal survival and death. J Biol Chem. 2012;287(42):35444–53. doi: 10.1074/jbc.M112.394544 22918830 10.1074/jbc.M112.394544 PMC3471765

[54] Graff J, Rei D, Guan J-S, Wang W-Y, Seo J, Hennig KM, et al An epigenetic blockade of cognitive functions in the neurodegenerating brain. Nature. 2012;483(7388):222–6. doi: 10.1038/nature10849 22388814 10.1038/nature10849 PMC3498952

[55] Akhtar MW, Raingo J, Nelson ED, Montgomery RL, Olson EN, Kavalali ET, et al Histone deacetylases 1 and 2 form a developmental switch that controls excitatory synapse maturation and function. J Neurosci. 2009;29(25):8288–97. doi: 10.1523/JNEUROSCI.0097-09.2009 19553468 10.1523/JNEUROSCI.0097-09.2009 PMC2895817

[56] Ferrari A, Fiorino E, Giudici M, Gilardi F, Galmozzi A, Mitro N, et al Linking epigenetics to lipid metabolism: Focus on histone deacetylases. Mol Membr Biol. 2012;29(7):257–66. doi: 10.3109/09687688.2012.729094 23095054 10.3109/09687688.2012.729094

[57] Mitro N, Godio C, De Fabiani E, Scotti E, Galmozzi A, Gilardi F, et al Insights in the regulation of cholesterol 7α-hydroxylase gene reveal a target for modulating bile acid synthesis. Hepatology. 2007;46(3):885–97. doi: 10.1002/hep.21819 17654698 10.1002/hep.21819

[58] Cantuti-Castelvetri L, Fitzner D, Bosch-Queralt M, Weil M-T, Su M, Sen P, et al Defective cholesterol clearance limits remyelination in the aged central nervous system. Science. 2018. 10.1126/science.aan4183 29301957

