# Supplementary figures and images for: Class I HDAC inhibition is a novel pathway for regulating astrocytic apoE secretion
Source: PLoS One. 2018 Mar 26;13(3):e0194661. doi: 10.1371/journal.pone.0194661 (PMC5868809; doi:10.1371/journal.pone.0194661)

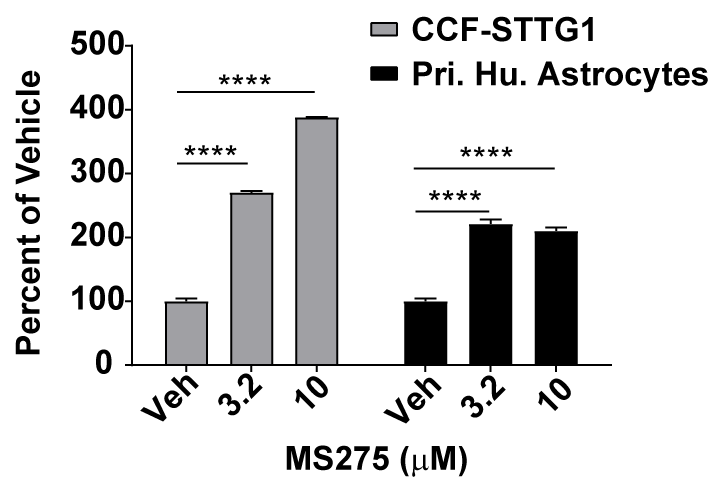

Supplement: S1 Fig — ApoE protein secretion from CCF-STTG1 cells and primary human astrocytes (lot number 06589) expressing apoE3/E4 genotype shows a concentration-dependent increase following treatment with MS275. Data were normalized to vehicle treated cells. All data are represented as values ± SEM with **** p<0.0001. (TIF) [file pone.0194661.s001.tif]

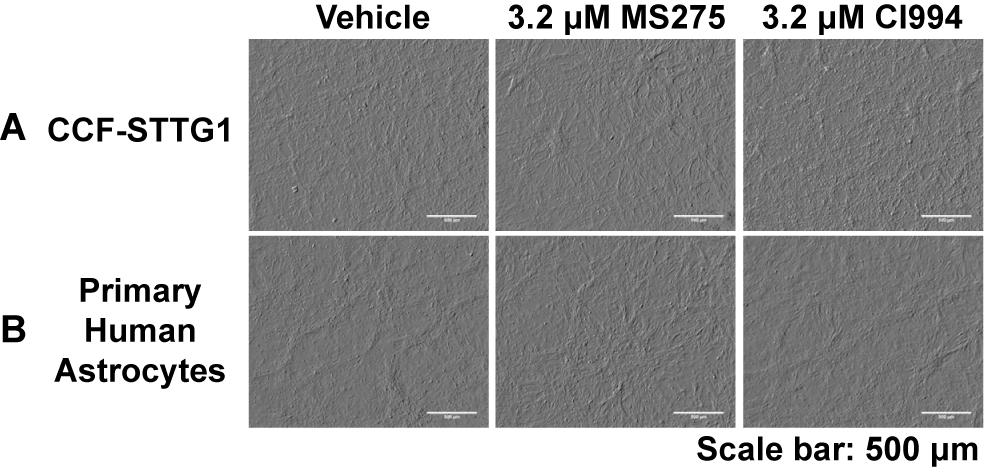

Supplement: S2 Fig — CCF-STTG1 cells and primary human astrocytes were imaged live 48hr post treatment with MS275, CI994, or vehicle. (A) CCF-STTG1 cells treated with vehicle (left), 3.2 μM MS275 (middle) and 3.2 μM CI994 (right). (B) Primary human astrocytes treated with vehicle (left), 3.2 μM MS275 (middle) and 3.2 μM CI994 (right). Scale bar = 500 μm. (TIF) [file pone.0194661.s002.tif]

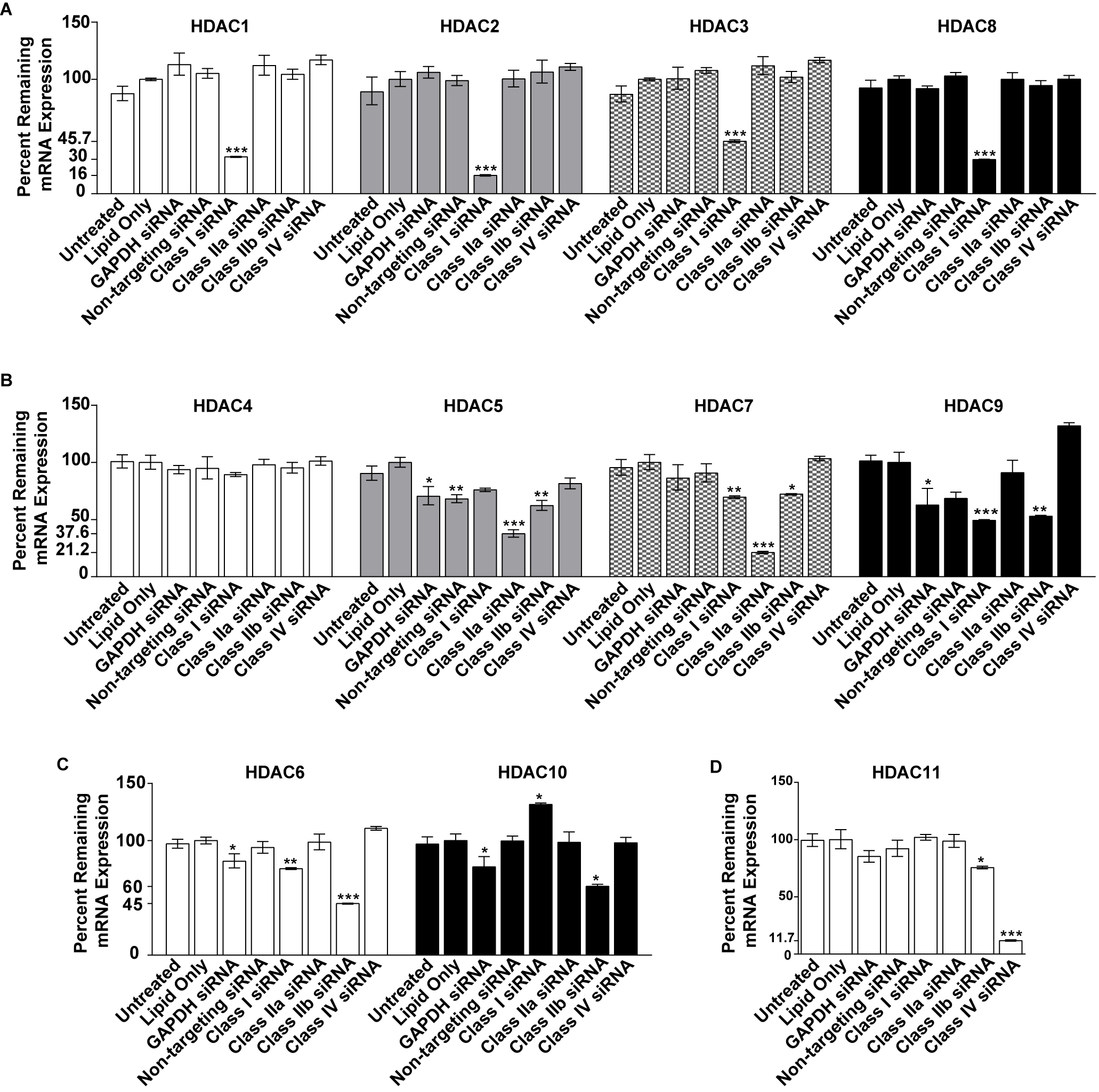

Supplement: S3 Fig — Each of the four classes of HDACs were knocked down using siRNA oligonucleotides in CCF-STTG1 cells and effect of knockdown on relative mRNA levels of each HDAC member was evaluated. The percent knockdown was calculated by normalizing the relative HDAC expression upon knockdown to the “lipid only” control. (A) Effect of knockdown on class I HDAC members. Significant reduction in the expression of class I HDACs (HDAC 1, 2, 3 and 8) was observed in response to pan class I HDAC knockdown. (B) Effect on class IIa HDAC members. Class IIa HDAC knockdown led to a significant decrease in HDAC 5 and 7 expression, but it had no effect on expression of HDAC 4 and 9. Interestingly, knocking down class I HDACs also decreased expression levels of HDAC 7 and 9. (C) Effect on class IIb HDAC members. Class IIb HDAC knockdown led to a decrease in expression of HDAC 6 and 10. (D) Effect on HDAC11, class IV HDAC member. Interestingly, a cross-over effect of knocking-down one class of HDAC on members belonging to the classes IIa, IIb and IV was also observed (B, C and D) suggesting interactions between the expression of various HDAC members. See S2 Table for siRNA oligonucleotides and S3 Table for gene expression assays. All data are represented as values ± SEM (n = 3); * p<0.05, ** p<0.01; *** p<0.001, student’s t test. (TIF) [file pone.0194661.s003.tif]

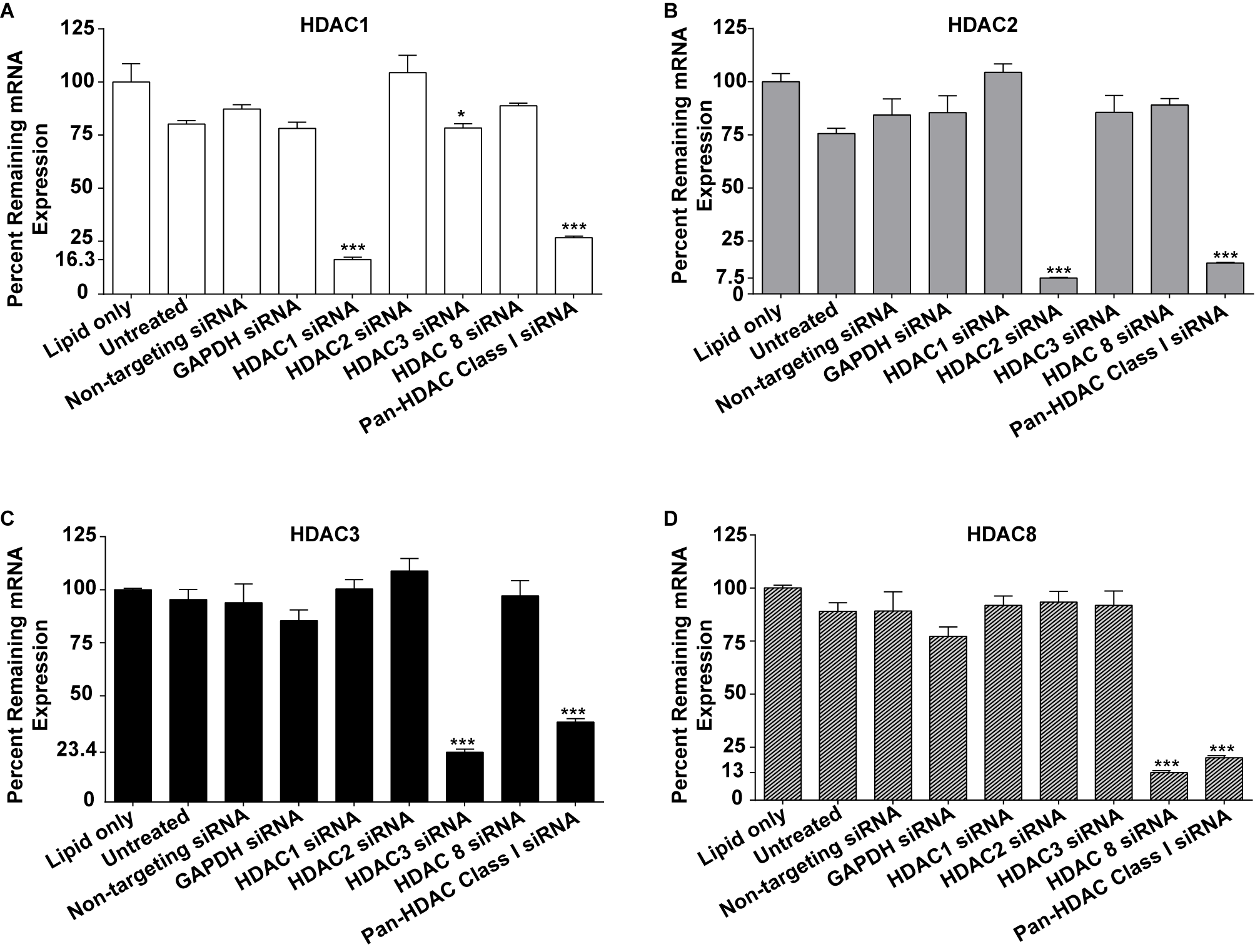

Supplement: S4 Fig — Each of the four members of pan class I HDAC were knocked down using a 40 nM concentration of pan class I HDAC siRNA or specific class I HDAC oligonucleotide in CCF-STTG1 cells grown in 6-well format (n = 3 per condition). Medium was replaced 24 hours post-transfection to serum free medium. Total RNA was extracted from cells 72 hours post-transfection and gene expression was evaluated using reverse transcription followed by quantitative real-time PCR. RNA expression was normalized to RPL13 mRNA expression. The percent knockdown was calculated by comparing with “lipid only” control. Knocking down using either an siRNA oligonucleotide targeting a specific member or using pan class I HDAC siRNA led to a significant decrease in the four members belonging to class I HDAC and is shown as follows: (A) HDAC1; (B) HDAC2; (C) HDAC3; and (D) HDAC8. Interestingly, knocking down HDAC3 led to a slight decrease in HDAC1 mRNA as shown in (A). See S2 Table for siRNA oligonucleotides and S3 Table for gene expression assays. All data are represented as values ± SEM with effect significance designated as follows: *** p<0.001. (TIF) [file pone.0194661.s004.tif]

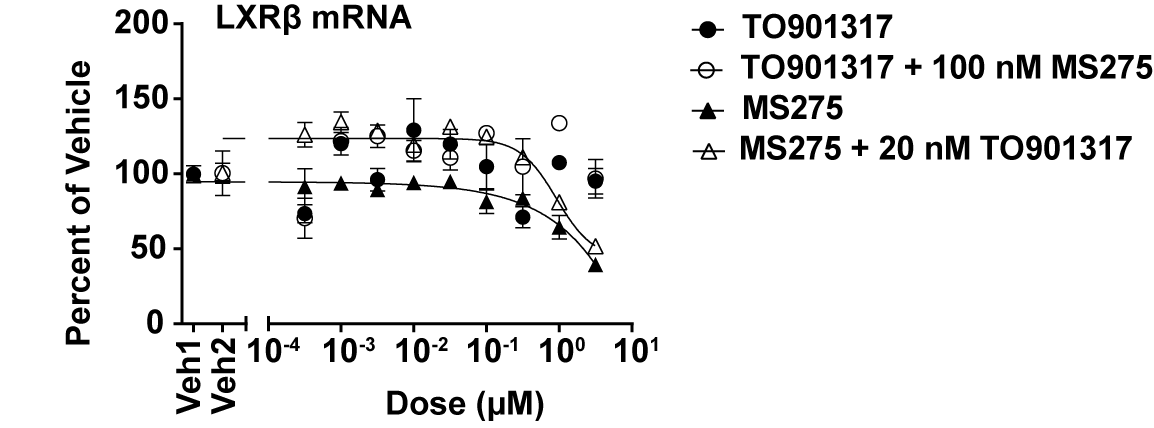

Supplement: S5 Fig — Treatment of TO901317 (●), MS275 (▲) or co-treatment of the compounds (TO901317 + 100 nM MS275 (o) or MS275 + 20 nM TO901317 (Δ)) on the levels of LXRβ mRNA. mRNA levels were normalized to vehicle treated controls (Veh1) and the effect of the constant compound (100 nM MS275 or 20 nM TO901317, denoted Veh2) was subtracted out after normalization. The average of GAPDH and 18S mRNA were used as endogenous controls for normalization of mRNA levels. See S3 Table for gene expression assays. All data are represented as values ±SEM. (TIF) [file pone.0194661.s005.tif]

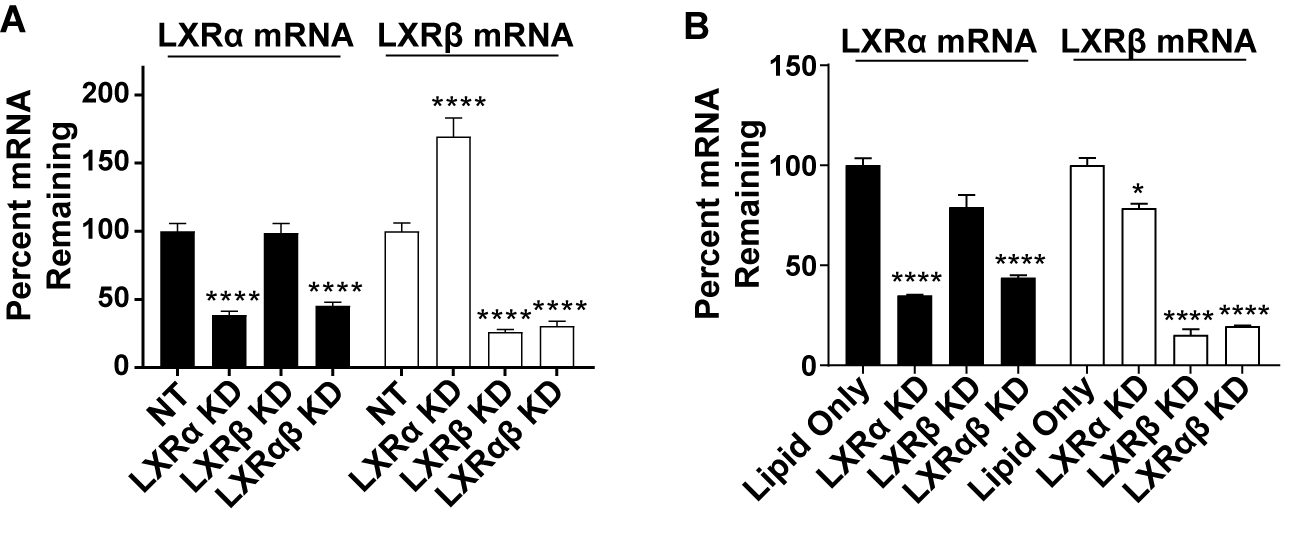

Supplement: S6 Fig — CCF-STTG1 cells (25,600 cells/well) or primary human astrocytes (22,500 cells/well) were grown in 96-well format and LXRα and LXRβ genes were knocked down either alone (LXRα KD or LXRβ KD) or in combination (LXRαβ KD) (n = 3 per condition). Total RNA was extracted and mRNA expression of LXRα and LXRβ genes were measured using quantitative real-time PCR and normalized to the average of GAPDH and 18s mRNA expression. See S2 Table for siRNA oligonucleotides and S3 Table for gene expression assays. (A) Significant knockdown (>55%) for LXRα and LXRβ genes was obtained when either was knocked down separately or in combination in CCF-STTG1 cells. (B) In primary human astrocytes (lot number 06589) greater than 60% knockdown was observed for LXRα and LXRβ genes both when knocked down separately or in combination. All data are represented as values ± SEM with effect significance designated as follows: * p<0.05, ****p <0.0001. (TIF) [file pone.0194661.s006.tif]

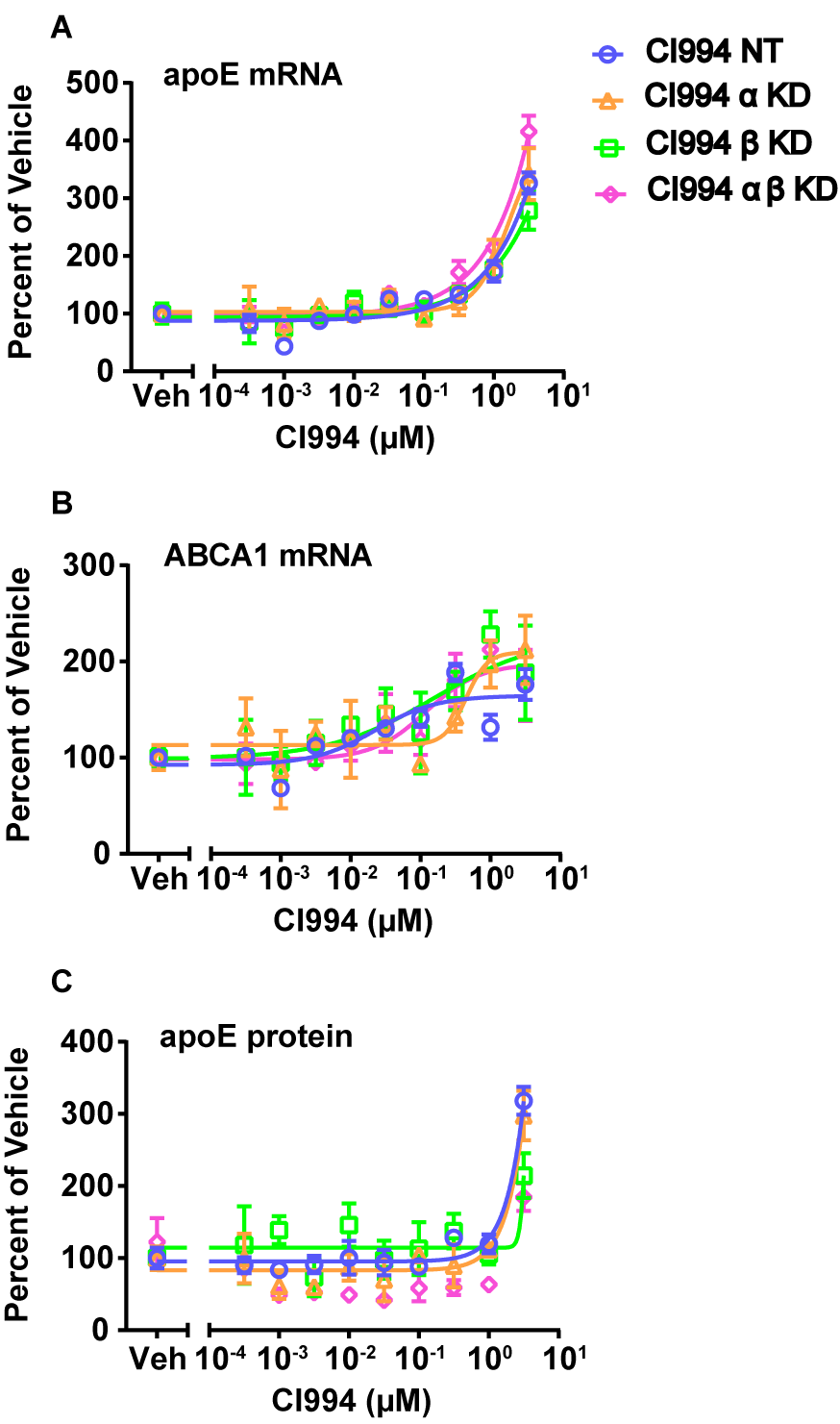

Supplement: S7 Fig — CCF-STTG1 cells were treated with the pan class I HDAC inhibitor, CI994, following knockdown of LXRα and LXRβ. (A) Concentration dependent effect on apoE mRNA levels following exposure with CI994 upon knocking down LXRα and LXRβ, separately or in combination. (B) Concentration dependent effect on ABCA1 mRNA levels following exposure with CI994 upon knocking down LXRα and LXRβ, separately or in combination. (C) Concentration dependent effect on apoE protein levels secreted into the media following exposure with CI994 upon knocking down LXRα and LXRβ, separately or in combination. See S2 Table for siRNA oligonucleotides and S3 Table for gene expression assays. All data are represented as values ± SEM. (TIF) [file pone.0194661.s007.tif]
