# Supplementary material for: Class I HDAC inhibition is a novel pathway for regulating astrocytic apoE secretion
Source: PLoS One. 2018 Mar 26;13(3):e0194661. doi: 10.1371/journal.pone.0194661 (PMC5868809; doi:10.1371/journal.pone.0194661)
Supplement: S1 Table — (DOCX) [file pone.0194661.s008.docx]

**S1 Table. Knock-down efficiency for pan class I HDACs.**

|  | **mRNA percent knockdown** | | | | **mRNA percent increase** |
| --- | --- | --- | --- | --- | --- |
| **Knockdown** | **HDAC1** | **HDAC2** | **HDAC3** | **HDAC8** | **apoE** |
| Pan-class I | 59 | 73 | 50 | 58 | 206 |
| Pan-class I | 66 | 81 | 49 | 62 | 222 |
