# Supplementary material for: Class I HDAC inhibition is a novel pathway for regulating astrocytic apoE secretion
Source: PLoS One. 2018 Mar 26;13(3):e0194661. doi: 10.1371/journal.pone.0194661 (PMC5868809; doi:10.1371/journal.pone.0194661)
Supplement: S2 Table — (DOCX) [file pone.0194661.s009.docx]

**S2 Table. siRNA oligos used in knock-down experiments.**

| **Target Gene** | **siRNA ID or Catalog No.** |
| --- | --- |
| HDAC1 | s73 |
| HDAC2 | s6493 |
| HDAC3 | s16878 |
| HDAC4 | s57940* |
| HDAC5 | s19462 |
| HDAC6 | s19459 |
| HDAC7 | s28335 |
| HDAC8 | s31698 |
| HDAC9 | s18773 |
| HDAC10 | s38333 |
| HDAC11 | s36566 |
| NR1H2 | s14684 |
| NR1H3 | s19568 |
| GAPDH | 4390849 |
| Negative Control No.1 | 4390843 |
| Negative Control No. 2 | 4390846 |
| Non-targeting pool No. 2 | D00120614 (Dharmacon) |

All siRNA oligos were purchased from Invitrogen unless otherwise noted

*This siRNA has been discontinued.
