# Supplementary material for: Class I HDAC inhibition is a novel pathway for regulating astrocytic apoE secretion
Source: PLoS One. 2018 Mar 26;13(3):e0194661. doi: 10.1371/journal.pone.0194661 (PMC5868809; doi:10.1371/journal.pone.0194661)
Supplement: S3 Table — (DOCX) [file pone.0194661.s010.docx]

**S3 Table. Gene expression assays for real time PCR.**

| **Gene** | **Assay ID** | **Amplicon Size (bp)** |
| --- | --- | --- |
| ABCA1 | Hs01059118_m1 | 61 |
| APOE | Hs00171168_m1 | 108 |
| NR1H3 | Hs00172885_m1 | 78 |
| NR1H2 | Hs01027215_g1 | 74 |
| HDAC1 | Hs00606262_g1 | 149 |
| HDAC1* | Hs02621185_s1 | 103 |
| HDAC2 | Hs00231032_m1 | 106 |
| HDAC3 | Hs00187320_m1 | 108 |
| HDAC4 | Hs01041648_m1 | 55 |
| HDAC4* | Hs01041638_m1 | 62 |
| HDAC5 | Hs00608366_m1 | 71 |
| HDAC6 | Hs00195869_m1 | 72 |
| HDAC7 | Hs00248789_m1 | 92 |
| HDAC8 | Hs00218503_m1 | 57 |
| HDAC8* | Hs00954353_g1 | 69 |
| HDAC9 | Hs00206843_m1 | 69 |
| HDAC10 | Hs00368899_m1 | 59 |
| HDAC11 | Hs00978041_m1 | 72 |
| GAPDH | Hs03929097_g1 | 58 |
| GAPDH* | Hs02758991_g1 | 93 |
| RN18S5 | Hs03928990_g1 | 61 |
| RPL13 | Hs00744303_s1 | 137 |

All gene expression assays were obtained from Applied Biosystems.

* Used only in HDAC expression profiling study
