# Supplementary material for: Class I HDAC inhibition is a novel pathway for regulating astrocytic apoE secretion
Source: PLoS One. 2018 Mar 26;13(3):e0194661. doi: 10.1371/journal.pone.0194661 (PMC5868809; doi:10.1371/journal.pone.0194661)
Supplement: S1 Text — (DOCX) [file pone.0194661.s011.docx]

**S1 Text. Pfizer chemogenomics library.**

The chemogenomics library developed at Pfizer was designed as a small screening set targeting known pharmacology to facilitate target deconvolution following a phenotypic screen. The set consists of 3,180 unique compounds covering 723 distinct biological mechanisms distributed into 11 gene classes. Criteria for inclusion in the chemogenomics library focused around two key parameters: potency against the target of interest and selectivity versus other targets for compounds within the Pfizer compound file. To maximize target coverage while allowing for sufficient dynamic range to show activity in a cellular assay at 10 µM, compounds with <500 nM potency against each target in the Pfizer database were identified and selectivity data (up to 10 µM for each measured endpoint) were recorded. The compound selection parameters used were as follows: activity against the target of interest, selectivity information (potency against other targets, number of assays, weighted violation score) and cellular potency. To aid in the selection of compounds, a Pareto multiparameter optimization function was developed taking into account the above criteria. The top five compounds for each target were selected to provide mechanism redundancy and build confidence in the involvement of a particular mechanism.
